# Nitrogen leaching and groundwater N contamination risk in saffron/wheat intercropping under different irrigation and soil fertilizers regimes

**DOI:** 10.1038/s41598-023-33817-5

**Published:** 2023-04-21

**Authors:** Mohammad Rasoul Abbasi, Ali Reza Sepaskhah

**Affiliations:** 1 Water Engineering Department, Shiraz University, Shiraz, Iran; 2 Drought Research Center, Shiraz University, Shiraz, Iran

**Keywords:** Environmental sciences, Environmental impact

## Abstract

The ever-rising trend of nitrate leaching from the agricultural production systems is a major risk to the contamination of ground- and surface-waters and should be addressed. But so far, there has been no study on the reduction of nitrate leaching from saffron fields through intercropping. Saffron growers can make a sustainable use of the saffron inter-row spaces through the strategy of winter-wheat/saffron base intercropping system to reduce nitrate leaching. During four years of study, in a set of lysimeters, effects of two cropping systems (saffron mono-cropping and saffron–wheat intercropping), application of two sources of nitrogen (organic cow manure and chemical granular urea) and four irrigation regimes [40, 60, 80, and 100% of the standard crop evapotranspiration (ET_c_)] on plant nitrogen and phosphorus uptake, nitrogen leaching and nitrogen and phosphorus efficiencies were investigated. The optimum irrigation regime was experienced at 60% ET_c_ (with irrigation application efficiency of 60%, equivalent to 100%ET_c_) where the highest saffron and wheat nutrient (nitrogen and phosphorus) uptake, nutrient (nitrogen and phosphorus) harvest indices, nutrient acquisition and use efficiencies, corm, saffron, and grain yields and lowest nitrogen loss was achieved. Moreover, manure application indicated 12, 42, 50 and 46% lower amounts of drained water, leachate nitrate nitrogen concentration, total leached nitrogen and N losses (other than N leaching), respectively, in comparison to the urea source of nitrogen showing the lower risk of groundwater nitrate pollution. Manure application showed 9, 8 and 9% increase in the concentration of corm nitrogen, phosphorus and protein, respectively, in comparison to urea application treatment. Saffron corm and stigma yields, irrigation and economic water productivities, corm nitrogen use efficiency and saffron-plant-nitrogen-acquisition efficiency in manure application surpassed respectively by 21, 25, 20, 17, 39 and 49% compared with the chemical source of nitrogen. Intercropping showed 10, 11, 23 and 64% lower amounts of drained water, nitrate concentration in drainage water, seasonal leached nitrate and N losses (other than N leaching), respectively compared to saffron sole cropping which reduces the risk of groundwaters nitrate contamination. For all the experimental treatments, empirical regression models were derived for estimation of seasonal leached nitrate based on the seasonal drained water. Intercropping saffron with winter wheat, application of organic cow manure and adopting irrigation regime of 60% ET_c_ is an innovative system of saffron production which mitigates the risk of groundwater nitrate contamination and increases irrigation and economic water productivities. Saffron growers can make sustainable and clean use of the inter-row spaces of the saffron crop to grow winter wheat in order to obtain higher economic water productivity and lower groundwater nitrate pollution, and it is highly recommended to maintain a sustainable environment.

## Introduction

Nitrogen element is critical to crop plants by involving in their growth, leaves expansion, reproduction and biomass-yield production processes ^1^. However, excessive application of nitrogen fertilizers has disturbed the global N cycle balance, resulting in major problems in environment, health, and economics. In a global scale, near 50% of the agricultural N fertilizers cannot be efficiently taken up and utilized by plants, and is lost in the environment in the forms of nitrate (NO_3_^−^), ammonia (NH_3_), and nitrous oxide (N_2_O), which increases the costs of agricultural production and contributes to water contamination and climate change ^2^. In recent years, the ever-rising trend of nitrate leaching due to the excessive use of N fertilizers in the agricultural sector combined with the improper irrigation regimes in such production systems has globally increased the risk of nitrate contamination in ground- and surface-waters ^3,4^. In semi-arid regions over-irrigation is another major cause of nitrate leaching ^5^. Nitrate concentration in potable groundwater supplies of Shiraz city which is located in a close vicinity of the experimental cite was in the range of 5 to 72 mg N L^−1^ which 11% of the potable groundwater samples had nitrate concentrations above the standard level of 10 mg N L^−1^ for drinking water. This was as a result of excessive use of chemical N fertilizers and manure in crop production systems resulting in nitrate leaching or due to the direct or indirect negative impacts of the industrial and municipal wastewaters on groundwater resources ^6^. This groundwater nitrate contamination is a growing anxiety posing significant threat to individuals, society and environment in this semi-arid region.

The above-mentioned N losses in crops production, and their environmental pollution potential has forced researchers to seek for easily adaptable agricultural management practices which can enhance N use efficiency and reduce N losses causing environmental deterioration. Some approaches have been developed to alleviate NO_3_^−^ leaching. The application of nitrification inhibitors has been indicated to be effective in reduction of nitrogen losses in grazed grassland ecosystems ^7^. Application of a nitrification inhibitor called nitrapyrin, maintained high NH_4_^+^/ NO_3_^−^ ratio in soil and thus decreased leaching of NO_3_^−^ in an intensive vegetable ecosystem ^8^. A number of studies attempted to determine the optimal value and type of nitrogen fertilizers to decrease N leaching ^9,10^. The use of some physical barrier materials, such as zeolite, was another strategy used to reduce NO_3_^–^ leaching ^11–13^. Some researchers tried to decrease N leaching through irrigation methods and strategies like partial root drying irrigation and water saving irrigation strategies ^5,14^. A few studies have conducted on using plants to reduce NO_3_^−^ leaching: Bergeron et al. ^15^ found that soil nutrient leaching reduced following the establishment of tree-based intercropping systems in eastern Canada. They found that tree roots in the tree-based intercropping system established on clay loam soil decreased subsoil NO_3_^–^ leaching by 227 kg N ha^−1^ and 30 kg N ha^−1^ over two consecutive years. In another survey, pepper/maize intercropping significantly reduced NO_3_^−^ leaching losses ^2^. However, there has been limited research on using crop plants to uptake N and reduce NO_3_^−^ leaching.

Saffron (*Crocus sativus* L.), the most precious spice in the world, is mainly used as food seasoning and coloring, in perfumes, cosmetics and medical purposes. It is cultivated in Iran as a leading country in saffron production (produces 90% of the world’s saffron) and a few other countries mostly located in arid and semi-arid regions ^16,17^ that facing nitrate pollution and shortages in irrigation water resources. Therefore, recommending a sustainable approach to reduce N leaching, enhancing nitrogen efficiencies and water productivity in saffron production areas is of particular importance. This help to plummet anthropogenic environmental degradation. Sharma et al. ^18^ developed a new mode of N placement, i.e., ‘mid rib placement upper to corms in two splits (MRPU‑2S)’ which could decrease nitrous oxide N emission by 70% and nitrate N leaching and runoff by 68 and 67%, respectively in comparison to conventional method, in saffron soils of the northwest Himalayas. In Iran, saffron corms are commonly cultivated in rows 0.25–0.35 m apart in basins ^16,19^. According to our hypothesis, saffron growers can make a sustainable use of the common saffron inter-row spaces through the novel strategy of wheat/ saffron-base intercropping under different sources of nitrogen which may result in higher plants’ (saffron and winter wheat plants) nitrogen uptake, irrigation and economic water productivities and N efficiency beside a mitigate in N leaching loss to the environment. In this system of cropping, winter wheat can be grown in parallel rows, each located between the saffron corm rows and aligned along them. Since the depth of irrigation water is a limiting key factor in N leaching management in this semi-arid region, therefore different irrigation water levels should also be examined to find an optimum irrigation regime appropriate to this system of cropping in this region that is facing water scarcity. Therefore, in the present investigation, for the very first time, during 4 years of study, the effects of saffron mono-cropping and saffron intercropped with winter wheat under different sources of nitrogen (chemical urea and organic manure) and different irrigation regimes [40, 60, 80 and 100% of the standard saffron evapotranspiration (ET_c_)] on plant nitrogen and phosphorus uptake, nitrogen leaching and nitrogen and phosphorus efficiencies were investigated in a semi-arid region of Iran (Fars province) in open-field lysimeters and their impacts on environment i.e. groundwater nitrate pollution, and total N loss were revealed. It should be noticed that in the mentioned saffron/wheat intercropping system, the amount of irrigation water for each irrigation event was calculated and adopted only based on water requirement of saffron crop and no extra water was applied for wheat crop. This gives the present study a greater importance as it provides more efficient use of irrigation water in the current water scarcity condition.

## Materials and methods

### Site description

This lysimeter study was conducted during the four consecutive growing seasons, 2013 to 2017 (2013–2014, 2014–2015, 2015–2016 and 2016–2017) at the Experimental Research Station of the Agricultural College, Shiraz University, Iran (29° 43' 44.0" N, 52° 35' 10.9" E, 1810 MSL) with the same experimental layout design in all the four growing seasons. The experimental site was located in a semi-arid region in southwest of Iran with a long-term average annual precipitation, relative humidity and air temperature of 387 mm, 52.2% and 13.4 °C, respectively. The mean monthly climatic data for the years of experiment are presented in Tables S1 and S2. Rainfall events were mostly occurred during November to May over the years of study as 279, 233, 289 and 368 mm for the first, second, third and fourth year, respectively. Higher precipitation depths took place in November and January of 2013–2014, November and March of 2014–2015, November, December and January of 2015–2016 and February and March of 2016-2017. The physico-chemical properties of the soil of lysimeters are presented in Table S3. The whole soil profile depth (Fine, mixed, mesic, Typic Calcixerepts) was classified as clay loam. The chemical analysis for the irrigation water is illustrated in Table S4 where there were no sodium and salinity hazards in the irrigation water.

### Lysimeters’ description

**Figure 1:**
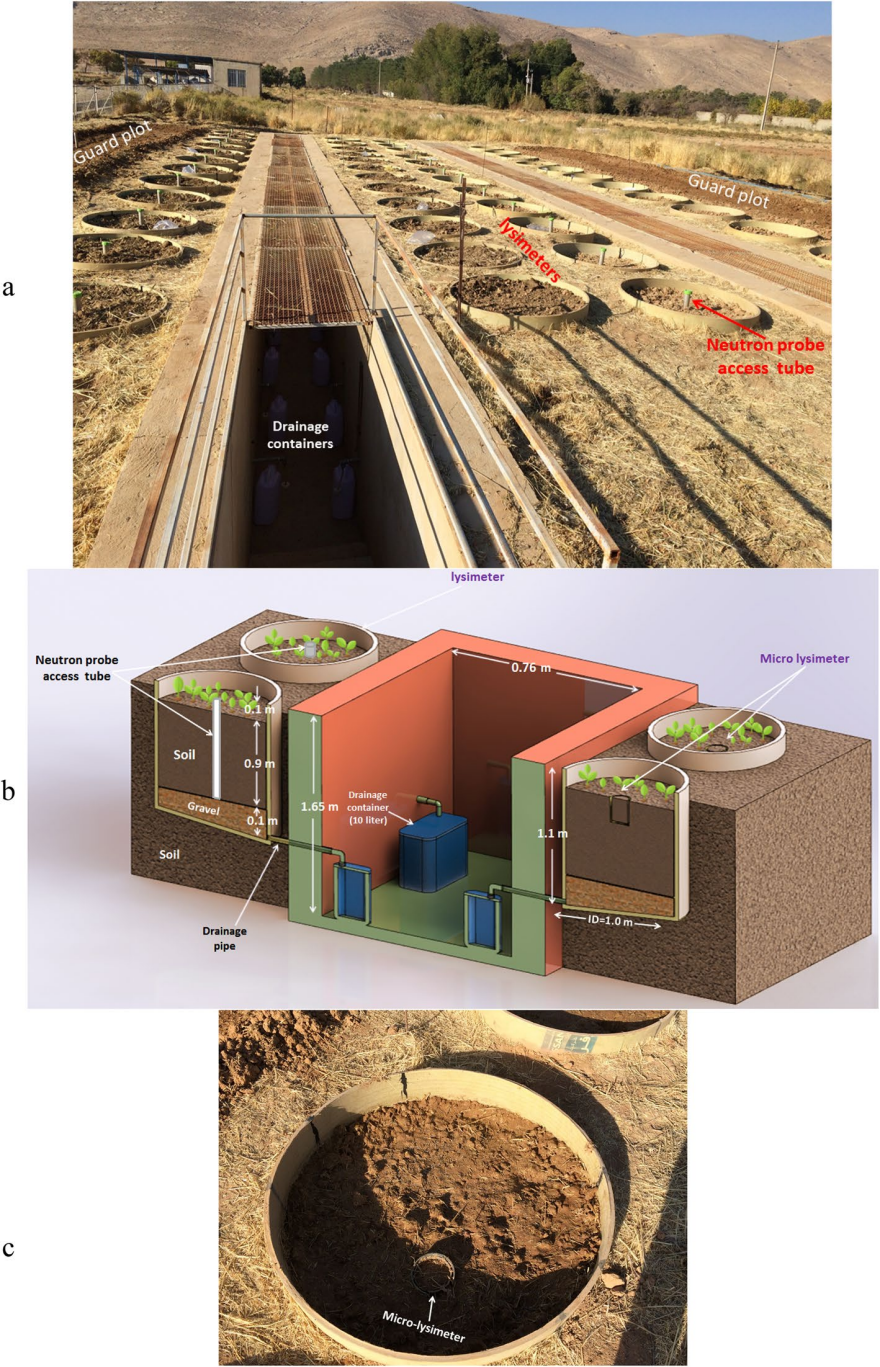
(**a**) A natural view of the in-field lysimeters' set. (**b**) A 3D schematic structure (cross-sectional view) of the in-field lysimeters' set. (**c**) A top view of a lysimeter and its micro-lysimeter.

This experiment was conducted in 48 in-field water balance GRP lysimeters (100-cm inner diameter and 110 cm length) (Farasan Manufacturing & Industrial Company, Iran, Fars Province, Shiraz) (Fig. 1a). The bottom of each lysimeters was blocked and water sealed slopping toward a drain pipe connected to a10-litter drainage container through a flexible drain tube (Fig. 1b). A layer of 0.1 m gravel as filter was placed at the bottom of each lysimeter and 0.90 m-thick soil layer was put on top. Two 15 m × 2 m guard plots were constructed along both sides of the set of lysimeters and winter wheat was planted inside to reduce the adjacent environmental influences. A number of 24 PVC micro-lysimeters (Fig. 1c) (105.6 mm inner diameter and 250-mm long) were installed into the soil of half the lysimeters and filled with surrounding soil and left exposed to environmental conditions to estimate evaporation from soil. To determine soil volumetric water content (SWC), 2-inch-diameter aluminum access tubes (1.0 m long) were installed at the center of half of the lysimeters (24 lysimeters) (Fig. 1b) and SWC was measured by a neutron scattering apparatus using a CPN 503DR hydroprobe (CPN Corp., Santa Barbara, CA).

### Experimental design

The experiment was conducted in a $2 \times 2 \times 4$ factorial arrangement of the treatments with three replications in a randomized complete block experimental design (RCBD) over four years comprised two systems of cropping (saffron mono-cropping and saffron intercropped with winter wheat), two different sources of nitrogen (fermented cow manure and chemical urea source of nitrogen) and four irrigation regimes [40, 60, 80, and 100% of the standard crop evapotranspiration (${\mathrm{ET}}_{\mathrm{c}}$)].

### Agronomic practices and measurements

In all the growing seasons, in late August, the top soil of each lysimeter was deeply plowed and triple superphosphate (100 kg P ha^−1^) was mixed with the soil and leveled. For the manure treatment, the top 0.15 m soil layer was supplied by 30 Mg ha^–1^ fermented cow manure as a source of nitrogen. The chemical characteristics of the fermented cow manure are presented in Table S5. On August 26 of the first growing season, semi big-sized saffron corms (> 8 g) were planted at the soil depth of 15–20 cm in three 30-cm-apart rows with a density of ${15\mathrm{ Mg ha}}^{-1}$ in each lysimeter. In each growing season, on October 29 to 30, winter wheat (Shiraz cultivar) seeds [250 kg ha^−1^ ^20^] were planted in the intercropping treatments at 5 cm soil depth in three parallel rows each located in the middle of the bare soil space between the rows of saffron corms (Fig. S1). Chemical urea treatments were supplied with granular urea fertilizer (120 kg N ha^−1^) half of which was applied just prior to the first irrigation immediately after the sowing of winter wheat at late October and the remaining split was applied at March (approximately 120–140 days after first irrigation). Over every November of all the growing seasons, during the flowering time, fresh flowers of all the treatments were manually picked up early every morning and the three-part stigmas and styles were separated from the stamens and petals. For each replication of any treatments, all the collected stigmas and styles were shadow-air dried in room condition for about 2–3 weeks and weights were measured precisely as saffron yield. At the end of each growing season, saffron leaves, wheat grain and straw were completely harvested from each lysimeter, oven dried, weighed and divided by its harvested area to determine saffron above-ground biomass, grain and straw yields, respectively. At the end of last (fourth) growing season, saffron corms were uprooted and corm yield was measured.

### Irrigation

Prior to each irrigation event, the volumetric soil water content (θ_i_) was measured at 0.3, 0.6 and 0.75 m of soil depths with a neutron scattering apparatus. Saffron gross water requirement (Eq. 1) was calculated based on increasing soil water content to the field capacity (one of the applied methods in this study) considering irrigation application efficiency of 60% (common irrigation efficiency applied by local farmers) ^16^:

1 $${d}_{g}=\frac{\sum_{i=1}^{n}\left({\theta }_{FCi}-{\theta }_{i}\right)\times {\Delta z}_{i}}{{E}_{a}}$$

where d_g_ is the gross irrigation water depth (m) for irrigation regime of 100% ET_c_, Δz_i_ is the soil thickness at layer i of saffron rooting depth (m), n is the number of soil layers in saffron rooting depth (RD), θ_FCi_ and θ_i_ are the volumetric soil water contents of layer i at field capacity and before irrigation (m^3^ m^−3^), respectively and E_a_ is irrigation application efficiency [decimal]. For the irrigation regimes of 80, 60 and 40% ET_c_, 80, 60 and 40% of the amount calculated in Eq. (1) was applied, respectively. For each growing season, saffron root depth was estimated using Eq. (2) suggested by Borg and Grimes ^21^:

2 $$RD= {RD}_{min}+{RD}_{max} \left[0.5+0.5\mathit{sin}\left(3.03\frac{DAFI}{DTM}- 1.47\right)\right]$$

where $\mathrm{RD}$ is the saffron rooting depth (m), ${\mathrm{RD}}_{\mathrm{min}}$ is the sowing depth of saffron corms (m), ${\mathrm{RD}}_{\mathrm{max}}$ is maximum root depth of saffron [0.45 m for saffron ^22^], $\mathrm{DAFI}$ is number of days after first irrigation which was reset for each growing season, $\mathrm{DTM}$ is the number of days after first irrigation event that root reaches its maximum depth [173 days for saffron ^23^]. However, for each growing season, to calculate saffron gross water requirement for the first irrigation event, a soil depth of 40 cm was considered instead of RD. The first irrigation of all the lysimeters was performed immediately after sowing of the winter wheat seeds in late October. Irrigation of intercropping treatments was carried out based on the saffron (main plant) irrigation amounts and interval and no extra water was applied for winter wheat crop. Over the periods of time with no adequate rain, irrigation interval of 24 days was adopted based on the saffron plant ^24^. According to this rule, 5, 6, 6 and 5 irrigation events were conducted for all the treatments in the first, second, third and fourth growing seasons, respectively. At the first growing season, in order to help a good establishment of saffron plants all the treatments were fully irrigated at the first irrigation event which was adopted in late October 2013, and afterwards the experimental irrigation regimes were carried out. Last irrigation was adopted in late April while saffron leaf senescence was initiating, and wheat plant growth continued without irrigation.

### Drainage water depth, its nitrogen concentration and leached nitrogen

The volume of the drained water collected from the bottom of each lysimeters were measured by a volumetric container after each irrigation event and divided by the area of the lysimeter (0.79 m) to get the drainage depth. After each irrigation event, the 0.1 L samples were taken from the drainage water of each lysimeter and kept at 4 °C, and its nitrate (NO_3_^−^) concentration was determined by spectrophotometer in less than 24 h after sampling. For each irrigation event, leached nitrate was obtained by multiplying the drainage water volume by its NO_3_^−^ concentration. For each growing season, seasonal leached nitrate was obtained from the sum of leached nitrate after each irrigation or rainfall event over that growing season. Finally, seasonal leached nitrogen was expresses as a percentage of the total applied nitrogen through manure or chemical urea fertilizer.

### Evapotranspiration and its components

The plant(s) actual evapotranspiration (ET_a_) for the irrigation regimes was estimated through the water balance method applying Eq. (3) ^16^:

3 $${ET}_{a}=I+P-D\pm \Delta S$$

where I is the irrigation depth [mm], P is the precipitation depth [mm], D is the drainage water depth [mm], and ΔS is soil water content change [mm] between two consecutive irrigation event in the root zone.

### Irrigation and economic water productivities

Irrigation water productivity (IWP) was calculated as crop yield per cubic meter of total water use, including rainfall and irrigation water ^25^ (Eq. 4).

Economic water productivity (EWP) was calculated as gross income in US$ per total water used in m^3^ ^26^ (Eq. 5).

4 $$IWP=\frac{Y}{ {W}_{Irr.}+{W}_{Rain}}$$

5 $$EWP=\frac{Y*P}{ {W}_{Irr.}+{W}_{Rain}}$$

where, IWP is irrigation water productivity for saffron (dry stigmas) or grain yields (kg m^−3^), Y is yield (saffron dry stigmas or wheat grain) (kg ha^−1^), W_Irr._ and W_Rain_ are irrigation and rain water use, respectively (m^3^ ha^−1^), P is the yield (saffron stigmas or wheat grain) price [US$ kg^-1^] and EWP is economic water productivity (US$ m^−3^) for saffron, grain or total yields. The prices of wheat and saffron were US$ 0.38 kg^−1^ and US$ 1066.7 kg^−1^, respectively ^27^. Saffron and wheat grain prices were in Iranian Rials and 1US$ = 300,000 Rials ^28^ was used to convert the prices from Iranian Rial to US Dollar.

### Laboratory measurements

For the last (fourth) growing season, saffron corm, saffron aboveground biomass (leaves and petals) and aboveground biomass of the winter wheat plant (grain and straw) were oven dried at 70 °C and their total nitrogen (N) and phosphorus (P) concentration were measured according to Kjeldahl and ammonium-vanadate-molybdate methods, respectively ^29^. The protein concentration of plant organs (saffron corm, saffron aboveground biomass, wheat grain and straw biomasses) were determined through multiplying its Kjeldahl nitrogen concentration by a Kjeldahl-nitrogen-to-protein conversion factor of 6.25 ^30^. Furthermore, the nitrate (NO_3_^−^) concentration of the drainage water was determined by spectrophotometer at 25 °C using a previously calibrated scanning spectrophotometer (JENWAY 6405 UV/Vis., Dunmow, Essex, UK) set at 220 and 275 nm where the absorbance at 275 nm was taken as the background in the two wavelengths determination method of nitrate. Leachate nitrate concentration was determined using a previously prepared standard nitrate curve ^31^.

### Nitrogen and phosphorus indicators

#### Nutrient (nitrogen and phosphorus) harvest indices

For wheat plant, nitrogen harvest index (NHI_Wheat_) is defined as the ratio between nitrogen uptake in grain (N_Grain_) and nitrogen uptake in grain plus straw (N_Grain_ + N_Straw_) multiplied by hundred ^32^ (Eq. 6). By a simple modification for saffron, the ratio between nitrogen uptake in corm (as the saffron plant’s main nitrogen sink) yield (N_Corm_) and nitrogen uptake in corm plus aboveground biomass yields (N_Corm_ + N_Abbvg._) multiplied by 100 would result in saffron plant nitrogen harvest index (NHI_Saffron_) (Eq. 7).

6 $${NHI}_{Wheat}=\frac{{N}_{Grain}}{ {N}_{Grain}+{N}_{Straw}}*100$$

7 $${NHI}_{Saffron}=\frac{{N}_{Corm}}{ {N}_{Corm}+{N}_{Abvg.}}*100$$

where, NHI_Wheat_ and NHI_Saffron_ are nitrogen harvest index for wheat and saffron, respectively (%), N_Grain_, N_Straw_, N_Corm_ and N_Abovg._ are nitrogen uptake by wheat grain, wheat straw, saffron corm and saffron aboveground biomass yields, respectively (kg ha^−1^).

By a simple modification, phosphorus harvest indices for wheat and saffron would be as Eqs. (8) and (9), respectively.

8 $${PHI}_{wheat}=\frac{{P}_{Grain}}{ {P}_{Grain}+{P}_{Straw}}*100$$

9 $${PHI}_{Saffron}=\frac{{P}_{Corm}}{ {P}_{Corm}+{P}_{Abvg.}}*100$$

where, PHI_Wheat_ and PHI_Saffron_ are phosphorus harvest index for wheat and saffron, respectively (%), P_Grain_, P_Straw_, P_Corm_ and P_Abovg._ are phosphorus uptake by wheat grain, wheat straw, saffron corm and saffron aboveground biomass yields, respectively (kg ha^−1^).

#### Nutrient (nitrogen and phosphorus) acquisition (uptake) efficiency

The nitrogen acquisition efficiency (NAE) is a soil-based nitrogen efficiency ^33^ which addresses the nitrogen uptake by yield (grain for wheat and corm for saffron) per unit of available nitrogen in soil system (the sum of soil initial available N and fertilizers’ available N). When flowering is finished at the first growing season, the daughter corms start to develop and grow on top of the mother corms. At the end of the first growing season, the color of saffron leaves change from green to yellow and development of the daughter corms is completed ^34^. At the following growing seasons with the aging of saffron plant, primary mother corms gradually become smaller and smaller. Hence, in this study, mother corm nitrogen content was not considered in NAE calculation for the fourth growing season. Therefore, grain and corm NAE is calculated as Eqs. (10) and (11) ^33^:

10 $${NAE}_{Grain}=\frac{{N}_{Grain}}{{N}_{Soil}+ {N}_{Fer.}}*100$$

11 $${NAE}_{Corm}=\frac{{ N}_{Corm}}{{N}_{Soil}+ {N}_{Fer.}}*100$$

where, NAE_Grain_ and NAE_Corm_ are nitrogen acqisition efficiencies of grain and corm (%), respectively, N_Grain_ and N_Corm_ are the grain and corm nitrogen uptake (kg ha^−1^), respectively, N_Soil_ and N_Fer._ are the soil and fertilizere (manure or chemical urea) available nitrogen (kg ha^−1^), respectively. For PAE calculations, phosphorus values have to be replaced with nitrogen in Eqs. (10) and (11). Moreover, NAE and PAE can be calculated for the whole plant. For example, NAE for saffron plant is the nitrogen uptake by saffron plant (corm and above-ground biomass) per unit of available nitrogen in soil system (soil plus applied fertilizer).

#### Nutrient (nitrogen and phosphorus) utilization efficiency

The nutrient (N and P) utilization efficiency (NUtE and PUtE, respectively) addresses the yield produced per unit of N and P, respectively, acquired (uptake) by the plant (Eqs. 12, 13) ^35^$.$

12 $${NUtE}_{Grain}=\frac{{Y}_{Grain}}{{N}_{Grain}+ {N}_{Straw}}*100$$

13 $${NUtE}_{Corm}={\frac{{Y}_{Corm}}{{N}_{Corm}+ N}}_{Abvg.}*100$$

where, NUtE_Grain_ and NUtE_Corm_ are grain and corm nitrogen utilization efficiencies (kg kg^−1^), respectively, Y_Grain_ and Y_Corm_ are the grain and corm yields (kg ha^−1^), respectively, N_Grain_, N_Straw_, N_Corm_ and N_Abvg._ are nitrogen uptake by whrat grain, wheat straw, saffron corm, saffron aboveground biomasses (kg ha^−1^), respectively. For PUtE calculations, phosphorus values have to be replaced with nitrogen in Eqs. (12) and (13).

#### Nutrient (nitrogen and phosphorus) use efficiency

Nitrogen use efficiency is a soil-based nitrogen efficiency and is defined as yield per unit of soil-system available nitrogen (the sum of soil initial available N and fertilizer available N) ^33,35^ as Eqs. (14) and (15):

14 $${NUE}_{Grain}=\frac{{Y}_{Grain}}{{N}_{Soil}+ {N}_{Fer.}}$$

15 $${NUE}_{Corm}=\frac{{ Y}_{Corm}}{{N}_{Soil}+ {N}_{Fer.}}$$

where, NUE_Grain_ and NUE_Corm_ are nitrogen use efficiencies of grain and corm (kg kg^−1^), respectively, Y_Grain_ and Y_Corm_ are the grain and corm yields (kg ha^−1^), respectively, N_Soil_ and N_Fer._ are the soil and fertilizere (manure or chemical urea) available nitrogen (kg ha^−1^), respectively. This definition can be simply modified for phosphorus use efficiency as yield per unit of P available in soil system (the sum of soil initial available P and fertilizer available P). For PUE calculations, phosphorus values have to be replaced with nitrogen in Eqs. (14) and (15).

#### System N balance index (SNBI)

The nitrogen balance index of a system (SNBI) is calculated as Eq. (16) ^36^:

16 $$SNBI={N}_{Input}-{N}_{Output}-\Delta Soil \, \mathrm{ total \, N}$$

where SNBI is the system N balance index, N_Input_ is the system measured nitrogen inputs including nitrogen supply in fertilizer (chemical urea or organic manure), nitrogen from irrigation water and rain N depositions, N_Output_ is the measured system nitrogen outputs including crop N removal (saffron corm, saffron aboveground biomass, wheat grain and straw biomass), N losses through N leaching and ∆Soil total N, is nitrogen change in soil, all in kg ha^−1^. In this study, SNBI shows N loses through NH4 volatilization, denitrification, gas emissions [NO_x_] and plant senescence which could not be determined directly in this study. Since this is a lysimetric study and the lysimeters are closed and water-sealed around systems, there would be no nitrogen loss through surface runoff and soil erosion to be taken into account in nitrogen loss calculations.

### Statistical analysis

Minitab 16.2.4 statistical software was applied to determine interaction effects of irrigation regimes, sources of nitrogen and cropping systems. Analysis of variance (ANOVA) was carried out according to Tukey test to determine statistically significant differences between the means at 5% probability level. It is also confirmed that all procedures were conducted in accordance to the relevant guidelines and regulations.

## Results and discussion

### Saffron and winter wheat yields

A short summary of the combined analysis of (ANOVA) and mean comparisons for saffron, corm, grain and wheat straw yields are presented in Table S6 and Table 1. Results indicated that saffron (dried stigmas and styles), corm and wheat straw yields did not significantly affect by irrigation regimes due to the use of low irrigation water application efficiency (60.0%). This resulted in high gross irrigation water depth plus rainfall that resulted in not significant difference in saffron, corm and wheat straw yields in different values of applied water depths. The highest wheat grain yield was harvested from 60% ET_c_. Chemical urea fertilizer decreased 20.5 and 17.5% saffron and corm yields, respectively in comparison to the manure treatments (Table 1). This decline might be due to the fact that chemical urea fertilizer only supplies nitrogen which may have been subjected to leach partly by applied excess gross water, whereas manure provides nitrogen and some micronutrients gradually which are essential for better saffron plant growth. Better top-soil chemical and physical properties such as higher water holding capacity, greater amount of humus and aeration porosity, higher diversity and biological activities of soil organisms in the manure treatments are other reasons for higher saffron and corm yields in manure treatment compared to the urea treatment. Donyanavard et al. ^37^, Amiri ^38^ and Koocheki et al. ^39^ also surveyed the effects of chemical and manure fertilizers on saffron yield and reported higher saffron yield in manure treatments in comparison to chemical fertilizers with the same reasoning. However, the chemical urea treatment experienced significantly greater grain and straw yields compared to the manure treatment (Table 1) most probably due to the higher amount of available nitrogen in urea treatments than manure. Furthermore, intercropping decreased 15.0 1nd 19.0% saffron and corm yields, respectively (Table S6 and Table 1) due to nutrient competition of winter wheat. More details on yield and yield components of saffron and winter wheat is available in Abbasi and Sepaskhah ^16^.

**Table 1:** Means for saffron (dried stigmas and styles), corm, wheat grain and wheat straw yields (SY, CY, WGY and WSY, respectively.

Treatments	SY, kg ha^−1^	CY, Mg ha^−1^	WGY, Mg ha^−1^	WSY, Mg ha^−1^
Irrigation regime				
40% ET_c_	3.088^a^*	8.466^a^	2.153^c^	8.013^a^
60% ET_c_	3.427^a^	10.308^a^	2.946^a^	9.234^a^
80% ET_c_	3.419^a^	9.698^a^	2.67^ab^	8.972^a^
100% ET_c_	3.138^a^	9.204^a^	2.429^bc^	8.545^a^
Source of nitrogen				
Manure	3.641^a^	10.321^a^	2.246^b^	8.042^b^
Urea	2.895^b^	8.517^b^	2.854^a^	9.339^a^
Cropping system				
Mono-cropping	3.526^a^	10.427^a^	2.550	8.691
Intercropping	3.009^a^	8.411^b^	–	–
Growing season				
1st	2.718^b^	–	2.579^ab^	8.649^ab^
2nd	3.332^a^	–	2.317^b^	8.149^b^
3rd	3.47^a^	–	n.d.^1^	n.d.
4th	3.552^a^	9.419	2.752^a^	9.275^a^

*Means followed by similar letters in each column for each factor and each trait are not significantly different at 5% level of probability according to Tukey test.

*n.d.*  not determined.

### Drainage water

A short summary of the combined analysis of variance (ANOVA) and mean comparisons for the drainage water depth and its expression as a percentage of applied gross water are presented in Table S7 and Table 2, respectively. There was a significant difference among irrigation regimes on drainage water. The lowest and highest drainage water and its percentage were observed in 40 and 100% ET_c_, respectively due to the lowest and highest gross water applied. Generally, the higher the gross water depth, the greater the drained water and its percentage, and vice versa were occurred. Moreover, the urea treatment indicated a significant greater amount of drained water (11.5%) in comparison to the manure treatment owing to a higher induced soil cracks and pathways in the urea treatments which increased the risk of preferential flow and groundwater contamination. Furthermore, intercropping showed a significant lower (10.0%) amount of drained water compared to mono-cropping due to the higher amount of evapotranspiration in intercropping system (Table 2). This is in line with the results of Shili-Touzi et al ^40^ which measured drainage water 11.0% lower in intercropped wheat and fescue than in wheat grown as a monoculture. There was a significant difference between the growing seasons on drainage water depth. The last two (third and fourth) growing seasons experienced the highest drained water from the bottom of the lysimeters in comparison to the first and second growing seasons, and this was due to the greater amount of the sum of the rainfall and gross irrigation water applied in these two years of the study (Table 2).

**Table 2:** Means for seasonal drainage water, gross irrigation, rainfall and actual evapotranspiration, ETa, depths.

Treatment	Gross irrigation, mm	Drainage water, mm	Drainage water, %	Rainfall, mm	ET_a_, mm
Irrigation regime					
40% ET_c_	254.9^d^*	2.18^d^	0.87^d^	290	558
60% ET_c_	382.5^c^	57.19^c^	15.21^c^	290	613
80% ET_c_	510.1^b^	216.34^b^	42.69^b^	290	578
100% ET_c_	637.4^a^	345.16^a^	54.26^a^	290	572
Source of nitrogen					
Manure	446.2^a^	146.80^b^	26.49^b^	290	590
Urea	446.2^a^	163.64^a^	30.02^a^	290	570
Cropping system					
Mono-cropping	446.2^a^	162.92^a^	29.89^a^	290	575
Intercropping	446.2^a^	147.52^b^	26.62^b^	290	586
Growing season					
1st	420^c^	145.86^b^	28.17^b^	280	559
2nd	478.3^a^	145.89^b^	24.04^c^	232	566
3rd	472.5^b^	163.21^a^	27.87^b^	281	594
4th	414.2^d^	165.91^a^	32.94^a^	368	601

*Means followed by similar letters in each column for each factor and each trait are not significantly different at 5% level of probability according to Tukey test.

### Nitrogen concentration of the drainage water

The results of combined analysis of variance (ANOVA) and mean comparisons for the average seasonal nitrate concentration in leachate (Table S8 and Table 3, respectively) shows a significant difference between irrigation regimes. The lowest and highest averages for the seasonal nitrate concentration were measured in 40 and 100% ET_c_ (1.74 and 2.64 mg N L^−1^, respectively) due to the lowest and highest gross water applied (Table 2), respectively. Generally, by an increase in irrigation water depth a significant rise in leachate nitrate concentration was observed and it implies the risk of groundwater nitrate contamination in higher irrigation levels (80 and 100%) and a decrease in plant available nitrogen in root zone as a result. The latter issue can be found out from the lower evapotranspiration and biomass values in higher irrigation levels (Tables 2 and 1, respectively). These results were pursuant to the findings of Jehan et al. ^41^, which concluded that deficit irrigation of 60% FC along with dairy manure resulted in more nitrate concentration in the upper 60 cm layer of soil where it can be further available for the crops. However, they measured maximum nitrate concentration at 90 cm soil depth at irrigation treatment of 80% FC, while under full irrigation, nitrate leached down to 120 cm of soil depth where it becomes unavailable to crops.

**Table 3:** Means for average seasonal nitrate concentration in drainage water, seasonal cumulative leached nitrate, and seasonal leached nitrogen as a percentage of total available nitrogen in the applied fertilizer (urea or manure).

Treatment	Leachate nitrate concentration, mg N L^−1^	Leached nitrate, kg N ha^−1^	Leached nitrate, %
Irrigation regime			
40% ET_c_	1.742^d^*	0.0391^d^	0.0286^d^
60% ET_c_	2.056^c^	1.2972^c^	0.9874^c^
80% ET_c_	2.351^b^	5.3892^b^	4.0042^b^
100% ET_c_	2.642^a^	9.8962^a^	7.3903^a^
Source of nitrogen			
Manure	1.618^b^	2.7856^b^	1.6009^b^
Urea	2.777^a^	5.5252^a^	4.6043^a^
Cropping system			
Mono-cropping	2.321^a^	4.6966^a^	3.5414^a^
Intercropping	2.074^b^	3.6142^b^	2.6639^b^
Growing season			
1st	2.201^b^	3.8879^b^	2.9122^b^
2nd	2.099^c^	3.8874^b^	2.9171^b^
3rd	2.183^b^	4.5021^a^	3.3299^a^
4th	2.307^a^	4.3442^a^	3.2513^a^

*Means followed by similar letters in each column for each factor are not significantly different at 5% level of probability according to Tukey test.

In addition, the manure treatment indicated approximately 42.0% lower amount of nitrate nitrogen concentration in leachate in comparison to the urea treatment (Table 3) owing to the fact that cow manure acts as a slow-nitrogen-release fertilizer and avoids high nutrient losses ^41^. Furthermore, intercropping showed nearly 11.0% lower amount of nitrate concentration in drainage water in comparison to mono-cropping due to the improved nutrient-use efficiency in intercropping systems. This is consistent with the higher amount of evapotranspiration and biomass production in intercropping system (Tables 2 and 1, respectively) which implies greater crop-nitrogen consumption. The highest and lowest amount of leachate nitrate-concentration (2.3 and 2.01 mg N L^−1^) was measured in the fourth and second growing seasons, respectively, due to the highest and lowest precipitation amounts (Table 2). The first and second growing seasons indicated no significant difference due to receiving almost equal amount of rainfall.

**Figure 2:**
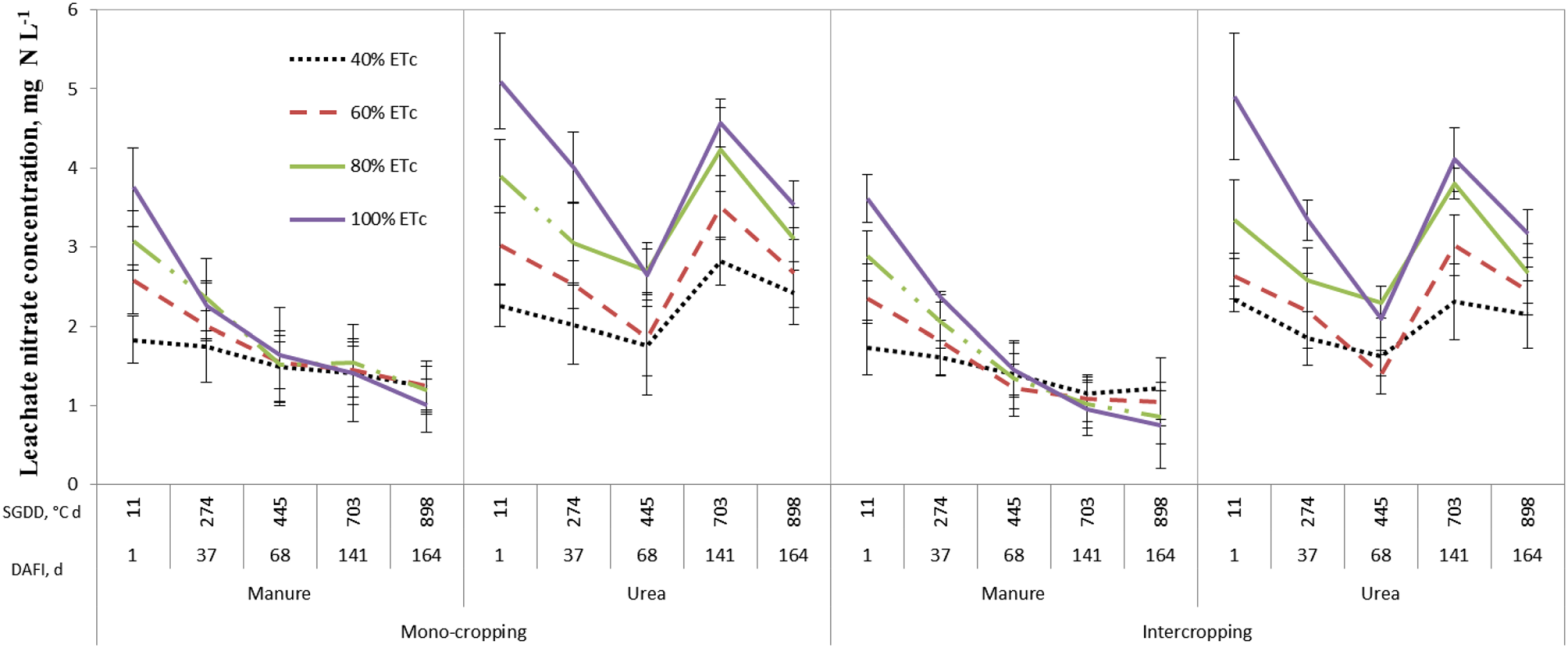
Mean nitrate concentration in the drainage water for different treatments based on both the saffron growing degree-day accumulation (SGDD) and days after first irrigation (DAFI) during the fourth growing season.

The changes in nitrate-nitrogen concentration of the drainage water for the fourth growing season are illustrated in Fig. 2. At the first irrigation event, 40 and 100% ET_c_ showed the lowest and highest leachate nitrate concentration due to lowest and highest leaching efficiencies, respectively. However, at the last irrigation event (164 DAFI), the place of the highest and lowest nitrate concentration of the leachate changed, that is 100 and 40% ET_c_ showed the lowest and highest leachate nitrate concentration in the manure treatments. This is again due to the higher leaching efficiency of the 100% ET_c_ in comparison to the lower levels of irrigation treatments. Overall, the leachate nitrate concentration of manure decreased over the whole growing season due to the leaching process and crop nitrogen use, while for urea treatments, after a decreasing trend, it rose and peaked at 141 days after first irrigation due to the application of the second split of urea fertilizer just before this date (the first half of crop required nitrogen was applied just before the first irrigation). Splitting nitrogen into two applications allows the plant to utilize N more efficiently. Generally, intercropping experienced lower values of leachate nitrate concentration in comparison to mono-cropping. The same trend was observed for other growing seasons. To make the manuscript brief, only the graphs of the fourth growing season is presented and compared.

### Nitrogen leaching

There was a significant difference between irrigation regimes on cumulative leached nitrate and its percentage of the total applied N (Table S8 and Table 3) and the trend is similar to nitrate concentration. The lowest and highest values for leached nitrate were observed in 40 and 100% ET_c_ (0.04 and 9.90 kg N ha^−1^, respectively) due to the lowest and highest volumes of applied water (Table 2), respectively. An increase in irrigation water depth resulted in a significant upward trend in leached nitrate concentration and the risk of groundwater contamination in higher irrigation levels (80 and 100%) and a decline in plant available nitrogen in root zone and lower plant growth (Table 1). These results was in line with the results of Jehan et al. ^41^, which found that deficit irrigation of 60% FC along with manure resulted in more nitrate concentration in the upper 60 cm soil layer. However, under full irrigation regime, nitrate leached down to 120 cm of the soil depth where it becomes unavailable to plant. Since cow manure is considered a slow-nitrogen-release fertilizer, the manure treatment experienced approximately 50.0% lower amount of total leached nitrogen in comparison to the urea treatment. Because of the improved nutrient-use efficiency in intercropping systems, intercropping showed nearly 23.0% lower amount of leached nitrate in leachate compared to mono-cropping (Table 3). This is consistent with the higher plant(s) biomass in intercropping system in comparison to mono-cropping (Table 1). Due to the higher total amount of irrigation and rainfall (Table 2), the last two growing seasons faced the highest leached nitrate from the bottom of the lysimeters in comparison to the first two years of the study. In other words, according to the leached nitrate percentage (Table 3) approximately 3.3 % of the fertilizers’ available nitrogen was washed out in the last two years compared to 2.9 % of the first 2 years.

Power relationships between cumulative leached nitrogen (L) and cumulative drained water (D) were derived for different treatments (Table 4). Coefficient and power of the variable measures the rate of change in the cumulative leached nitrate-nitrogen as the cumulative drainage water changes. The coefficient and power of equations for urea treatment were generally greater than those of manure treatment. Moreover, mono-cropping indicated a higher power, coefficient or both compared to intercropping treatment. The greater the power and coefficient, the steeper the line and the greater change in the leached nitrate per unit change in the drainage water. These equations are useful and practical to predict cumulative leached nitrate based on the cumulated drained water.

**Table 4:** The relationship between cumulative leached nitrogen (L) in kg N ha^-1^ and cumulative drained water (D) in mm.

Cropping system	Source of nitrogen	Irrigation regime	Power regression equation	R-square, %	n
Mono-cropping	Manure	0.4 ET_c_	L = 0.0158 D^0.938^	98.7	22
		0.6 ET_c_	L = 0.0295 D^0.883^	98.7	22
		0.8 ET_c_	L = 0.0731 D^0.7551^	98.9	22
		1.0 ET_c_	L = 0.1251 D^0.6988^	97.2	22
	Urea	0.4 ET_c_	L = 0.0219 D^0.947^	99.6	22
		0.6 ET_c_	L = 0.0431 D^0.8909^	98.6	22
		0.8 ET_c_	L = 0.0739 D^0.8474^	98.6	22
		1.0 ET_c_	L = 0.1275 D^0.7931^	98.7	22
Intercropping	Manure	0.4 ET_c_	L = 0.0148 D^0.9161^	98.8	22
		0.6 ET_c_	L = 0.0269 D^0.8753^	95.5	22
		0.8 ET_c_	L = 0.0927 D^0.6896^	96.2	22
		1.0 ET_c_	L = 0.1576 D^0.6399^	95.8	22
	Urea	0.4 ET_c_	L = 0.0204 D^0.9548^	99.4	22
		0.6 ET_c_	L = 0.033 D^0.9225^	97.5	22
		0.8 ET_c_	L = 0.0979 D^0.7646^	97.6	22
		1.0 ET_c_	L = 0.1168 D^0.7801^	98.6	22

### Water productivity

A short summary of combined analysis of variance (ANOVA) and mean comparisons for the irrigation and economic water productivities of saffron and winter wheat grain are presented in Table S9 and Table 5, respectively.

**Table 5:** Means for saffron and wheat-grain physical water productivities (WP_Saffron_ and WP_Grain_, respectively), saffron, wheat grain and total economic water productivities (EWP_Saffron_, EWP_Grain_ and EWP_Total_, respectively).

Treatments	WP_Saffron_ × 10^3^, kg m^−3^	WP_Grain_, kg m^−3^	EWP_Saffron_, US $ m^−3^	EWP_Grain_, US $ m^−3^	EWP_Total_, US $ ^−3^
Irrigation regime					
40% ET_c_	0.5684^a^*	0.3964^ab^	0.6063^a^	0.15196^ab^	0.6832^a^
60% ET_c_	0.5068^b^	0.4412^a^	0.5406^b^	0.16913^a^	0.6112^ab^
80% ET_c_	0.4274^c^	0.3366^b^	0.4559^c^	0.12903^b^	0.513^c^
100% ET_c_	0.3376^d^	0.2648^c^	0.3601^d^	0.10149^c^	0.4068^d^
Source of nitrogen					
Manure	0.5117^a^	0.3179^b^	0.5458^a^	0.12184^b^	0.5979^a^
Urea	0.4084^b^	0.4016^a^	0.4356^b^	0.15396^a^	0.5093^b^
Cropping system					
Mono-cropping	0.4958^a^	–	0.5288^a^	–	0.5166^b^
Intercropping	0.4244^a^	–	0.4527^b^	–	0.5905^a^
Growing season					
1st	0.405^b^	0.3797^a^	0.432^b^	0.14557^a^	0.5048^b^
2nd	0.492^a^	0.3385^a^	0.5248^a^	0.12974^a^	0.5896^a^
3rd	0.4773^a^	n.d.	0.5091^a^	n.d.	n.d.
4th	0.466^a^	0.361^a^	0.4971^a^	0.1384^a^	0.5663^a^

*Means followed by similar letters in each column for each factor and each trait are not significantly different at 5% level of probability according to Tukey test.

*n.d.*  not determined.

### Irrigation water productivity (IWP)

A significant difference in saffron irrigation water productivity was observed among the irrigation regimes (Table S9 and Table 5). Since there was no significant difference among irrigation regimes regarding to the saffron yield (Table 1), the only factor which makes this significant difference is the applied gross water depth (Table 2). The highest and lowest values for this index (IWP) achieved in 40 and 100% ET_c_, respectively due to the minimum and maximum amounts of the applied gross irrigation water, respectively. However, 60% ET_c_ experienced the highest wheat grain IPW since the highest grain yield was harvested from this irrigation regime (Table 1). The lowest grain IWP was fulfilled in 100% ET_c_ due to the greatest applied gross water depth (Table 2). In addition, irrigation water productivity in manure surpassed 20.0% the chemical source of nitrogen due to the 25.0% higher yield of saffron (Table 1), 11.0% lower drained water (Table 2) and 50% lower leached nitrate (Table 3) in manure treatment compared to urea fertilizer. A reversed outcome was achieved for grain IWP. That is to say, IWP for grain was 26.0% higher in chemical nitrogen application thanks to the higher yield of grain in urea fertilizer treatments. This is almost similar to IWP obtained for quinoa as 19.0% in N application rate of 100 kg ha^−1^ compared with that obtained in no N application ^42^. In contrast, IWP of quinoa has not been affected by soil organic amendment as reported by Hirich et al. ^43^.

Furthermore, there was no significant difference on saffron IPW between the cropping systems (Table S9 and Table 5) owing to a non-significant difference in saffron yield in these two systems of cropping (Table 1). The minimum of saffron IWP happened in the first growing season due to the lowest saffron yield in this year of study (Table 1) while the other growing seasons showed no significant difference regarding to this index. However, no significant difference was observed among the growing seasons in wheat grain IWP.

### Economic water productivity (EWP)

Economic water productivity (EWP) indicates how much economic outcome is produced per cubic meter of water applied. It serves as a measure of the efficiency of the used water. The trend and pattern of changes in EWP for saffron and grain yields (Table S9 and Table 5) was similar to that of the IWP which just discussed in “Irrigation water productivity (IWP)”. The highest total EWP achieved in 40% ET_c_, and 60% ET_c_ gained the second high rank. However, it decreased down significantly by an increase in the applied water depth. The total EWP of manure treatment was 17.0% greater than that of chemical source of nitrogen. Furthermore, intercropping showed a 14.0% higher value for total EWP which justifies the economic preference of saffron–wheat intercropping in comparison to saffron sole cropping. Furthermore, the effect of growing season on total EWP was significant and the first growing season experienced the lowest total EWP due to the lowest saffron yield (Table 1). However, there was not a significant difference between the second and last growing seasons according to total EWP.

### Nitrogen (N), phosphorus (P) and protein concentration in saffron and winter wheat organs

Irrigation regime did not have any significant influence on nitrogen, phosphorus (Table S10 and Table 6) and protein concentration (Table S11 and Table 7) of the saffron and winter wheat organs. However, manure showed 9.0, 8.0 and 9.0% increase in the concentration of corm nitrogen, phosphorus and protein, respectively, in comparison to urea treatment. This result might be due to the higher supply of organic matter and more balanced nutrients availability in manure ^44,45^ that can improve the nutritional conditions for the growth of corms ^38^ and therefore, a significant increase in saffron yield. Hence, it can be concluded that the application of chemical fertilizer of urea has less effect on corm growth. These results were in line with the findings of Koocheki and Seyyedi ^46^ who achieved a higher concentration of corm nitrogen and phosphorus in manure compared to application of chemical fertilizer. Howbeit, nitrogen, phosphorus and protein concentration in saffron above-ground portion and wheat above-ground plant yield components (grain and straw) was not affected significantly by source of nitrogen, although a minor increase could be distinguished in the manure treatment regarding to these elements. Moreover, except for corm nitrogen and protein concentration which was affected by intercropping, there was no significant difference in N and P concentration of saffron above-ground portion and corm phosphorus in sole cropping and intercropping. The 5% decrease in both nitrogen and protein concentration of saffron corms might be a consequence of the nutrition competition of wheat rooting system.

**Table 6:** Means for nitrogen and phosphorus concentration in the saffron and winter wheat organs for the last (fourth) growing season.

Treatments	Nitrogen concentration, %				Phosphorus concentration, g kg^−1^			
	Saffron		Wheat		Saffron		Wheat	
	Corm	Aboveground biomass	Grain	Straw	Corm	Aboveground biomass	Grain	Straw
Irrigation regime								
40% ET_c_	1.136^a^	0.90^a^*	1.47^a^	0.36^a^	2.04^a^	1.86^a^	2.30^a^	0.37^a^
60% ET_c_	1.22^a^	0.81^a^	1.61^a^	0.39^a^	2.20^a^	1.99^a^	2.51^a^	0.37^a^
80% ET_c_	1.18^a^	0.87^a^	1.55^a^	0.37^a^	2.13^a^	1.83^a^	2.33^a^	0.35^a^
100% ET_c_	1.15^a^	0.88^a^	1.42^a^	0.34^a^	2.04^a^	1.69^a^	2.28^a^	0.35^a^
Source of nitrogen								
Manure	1.22^a^	0.87^a^	1.51^a^	0.37^a^	2.18^a^	1.86^a^	2.44^a^	0.37^a^
Urea	1.12^b^	0.85^a^	1.52^a^	0.36^a^	2.03^b^	1.83^a^	2.27^a^	0.35^a^
Cropping system								
Mono-cropping	1.20^a^	0.89^a^	–	–	2.16^a^	1.84^a^	–	–
Intercropping	1.14^b^	0.84^a^	–	–	2.05^a^	1.84^a^	–	_–_

*Means followed by similar letters in each column for each factor and each trait are not significantly different at 5% level of probability according to Tukey test.

**Table 7:** Means for protein concentration (PC) of saffron corm, saffron plant aboveground biomass portion, winter wheat grain and straw in the last (fourth) growing season.

Treatments	Protein concentration, %			
	Saffron		Wheat	
	Corm	Aboveground biomass	Grain	Straw
Irrigation regime				
40% ET_c_	7.102^a^*	5.595^a^	9.195^a^	2.222^a^
60% ET_c_	7.6^a^	5.039^a^	10.0856^a^	2.428^a^
80% ET_c_	7.373^a^	5.424^a^	9.6598^a^	2.332^a^
100% ET_c_	7.155^a^	5.516^a^	8.8797^a^	2.128^a^
Source of nitrogen				
Manure	7.621^a^	5.452^a^	9.4299^a^	2.31^a^
Urea	6.994^b^	5.335^a^	9.4801^a^	2.245^a^
Cropping system				
Mono-cropping	7.498^a^	5.531^a^	–	–
Intercropping	7.117^b^	5.257^a^	–	–

*Means followed by similar letters in each column for each factor and each trait are not significantly different at 5% level of probability according to Tukey test.

### Crop(s) nutrient (nitrogen and phosphorus) uptake

Except for saffron corms, irrigation regimes did not affect nitrogen uptake by other organs of the plant(s) (saffron aboveground biomass, wheat grain and straw) (Table S12 and Table 8). The highest corm N and P uptake of 62.6 and 11.4 kg ha^−1^, respectively, was achieved in 60% ET_c_, where saffron yield was relatively maximized (Table 1). Furthermore, the results revealed that cow manure application was approximately 30.0% and 26.0% more efficient (Table 8) in increasing N and P uptake of saffron plant compared to the chemical N fertilizer due to higher nitrogen and phosphorus concentrations (Table 6). This finding is in accordance with those of Koocheki and Seyyedi ^46^ who reported a more pronounced higher nitrogen and phosphorus uptake by saffron corms in composted cattle manure compared to the chemical nitrogen. As mentioned before, this might be due to supplying organic matter and more balanced availability of nutrients in organic cow manure which might have improved the nutritional conditions for the corms growth. Unlike saffron, urea fertilizer increased nitrogen and phosphorus uptake by wheat grain compared to organic fertilizer due to the higher grain yield (Table 1). This refers to the fact that wheat N requirement is higher than that of saffron. This is in line with the findings of Shah and Ahmad ^47^ and Das et al. ^48^, which in the latter survey an approximately 20.0% higher wheat grain yield achieved in urea treatments (120 kg N ha^−1^) in comparison to farmyard manure in cotton–wheat cropping system. Moreover, intercropping lowered saffron nutrient (N and P) uptake in comparison to sole saffron due to the lower saffron nutrient concentration (Table 6) as a result of nutrient competition of wheat plant in saffron–wheat intercropping system.

**Table 8:** Means for nutrient (nitrogen and phosphorus) uptake by saffron (corm and aboveground) and winter wheat (grain and straw) biomasses and nitrogen and phosphorus harvest indices for saffron and wheat, all for the last (fourth) growing season.

Treatments	Nitrogen uptake, kg ha^−1^				Phosphorus uptake, kg ha^−1^				Nitrogen harvest index (NHI), %		Phosphorus harvest index (PHI), %	
	Saffron		Wheat		Saffron		Wheat		Saffron	Wheat	Saffron	Wheat
	Corm	Aboveground biomass	Grain	Straw	Corm	Aboveground biomass	Grain	Straw				
Irrigation regime												
40% ET_c_	48.1^b^*	16.7^a^	35.5^a^	30.7^a^	8.6^b^	3.4^a^	5.5^b^	3.1^a^	74.1^a^	53.3^a^	71.1^a^	63.3^a^
60% ET_c_	62.6^a^	17.3^a^	51.2^a^	37.5^a^	11.4^a^	4.3^a^	7.8^a^	3.6^a^	78.1^a^	57.4^a^	72.2^a^	68.5^a^
80% ET_c_	56.9^ab^	18.47^a^	44.5^a^	35.0^a^	10.2^ab^	3.9^a^	6.7^ab^	3.3^a^	75.0^a^	55.6^a^	72.0^a^	66.3^a^
100% ET_c_	52.1^ab^	17.4^a^	36.5^a^	31.5^a^	9.3^ab^	3.3^a^	5.9^ab^	3.2^a^	75.0^a^	54.2^a^	73.4^a^	64.9^a^
Source of nitrogen												
Manure	62.3^a^	19.3^a^	36.3^b^	30.9^a^	11.1^a^	4.1^a^	5.9^b^	3.0^a^	76.1^a^	54.1^a^	72.8^a^	65.6^a^
Urea	47.5^b^	15.6^b^	47.5^a^	36.4^a^	8.6^b^	3.4^b^	7.0^a^	3.6^a^	75.0^a^	56.2^a^	71.6^a^	66.0^a^
Cropping system												
Mono-cropping	62.2^a^	20.0^a^	–	–	11.2^a^	4.2^a^	–	–	75.4^a^	–	72.6^a^	–
Intercropping	47.6^b^	14.9^b^	–	–	8.5^b^	3.3^b^	–	–	75.7^a^	–	71.8^a^	–

*Means followed by similar letters in each column for each factor and each trait are not significantly different at 5% level of probability according to Tukey test.

## Nitrogen and phosphorus indicators

### Nutrient (nitrogen and phosphorus) harvest indices

There was no significant difference in terms of nitrogen and phosphorus harvest indices (NHI and PHI) for saffron and wheat plants among irrigation regimes, sources of nitrogen and cropping systems (Table S12 and Table 8). The values of NHI and PHI were higher than those of Koocheki and Seyyedi, ^46^ who reported the above-mentioned indices for saffron plant in the first 2 years of its life cycle and in different corm nutrient condition (25 t ha^−1^ cattle manure and 300 kg N ha^−1^ in chemical fertilizer) while we calculated them for the fourth year of corm life cycle with the application of 30 t ha^−1^ cow manure and 120 kg N ha^−1^ in manure and urea treatments, respectively. The greater NHI and PHI indicate directly the higher allocation of biomass nitrogen and phosphorus to grain. Indirectly, NHI or PHI indicate the way of partitioning between nitrogen or phosphorus uptake by grain and straw and NHI or PHI values allow for convenient prediction of straw nitrogen or phosphorus as grain nitrogen or phosphorus data are readily available.

### Nutrient acquisition (uptake) efficiency

Although there was not a high significance difference among the irrigation regimes, 60% ET_c_ experienced the highest nitrogen and phosphorus acquisition efficiencies, both in yield and in the whole plant (Table S13 and Table 9) which indicates the highest uptake efficiencies for P and N minerals. Furthermore, there was no significant difference between sources of nitrogen on NAE for grain and wheat plant and on PAE for corm and saffron plant. However, manure increased 51.0% and 49.0% nitrogen uptake efficiencies for corm and saffron plant, respectively, while for PAE of the grain and the whole wheat plant, the trend was opposite and urea fertilizer increased phosphorus uptake efficiency in wheat grain and in the whole wheat plant compared to animal manure. This is due to lower total available phosphorus in the urea treatment. In general, intercropping decreased yield and plant nutrient (P and N) uptake efficiencies in contrast to sole saffron due to the nutrient competition of wheat plant.

**Table 9:** Means for nitrogen and phosphorus acquisition efficiencies (NAE and PAE, respectively) for yield (corm or grain) and the whole plant (saffron and wheat), all for the last (fourth) growing season.

Treatments	Acquisition efficiency (for yield), %				Acquisition efficiency (for whole plant), %			
	NAE		PAE		NAE		PAE	
	Corm	Grain	Corm	Grain	Saffron	Wheat	Saffron	Wheat
Irrigation regime								
40% ET_c_	34.14^b^*	24.78^a^	5.05^b^	3.32^b^	46.01^b^	46.20^b^	7.07^b^	4.90^b^
60% ET_c_	44.25^a^	35.62^a^	6.73^a^	4.74^a^	56.48^a^	61.83^a^	9.25^a^	6.53^a^
80% ET_c_	40.44^ab^	30.95^a^	6.02^ab^	4.07^ab^	53.49^ab^	55.43^ab^	8.35^ab^	5.74^ab^
100% ET_c_	36.97^ab^	25.41^a^	5.46^ab^	3.56^ab^	49.29^ab^	47.34^b^	7.43^b^	5.16^b^
Source of nitrogen								
Manure	46.85^a^	27.31^a^	5.78^a^	3.05^b^	61.39^a^	50.55^a^	7.91^a^	3.95^b^
Urea	31.05^b^	31.06^a^	5.86^a^	4.79^a^	41.25^b^	54.85^a^	8.14^a^	7.22^a^
Cropping system								
Mono-cropping	44.13^a^	–	6.60^a^		58.30^a^	–	9.07^a^	–
Intercropping	33.77^b^	–	5.04^b^		44.33^b^	–	6.99^b^	–

*Means followed by similar letters in each column for each factor and each trait are not significantly different at 5% level of probability according to Tukey test.

### Nutrient utilization efficiency

Irrigation regimes showed no significant effect on nitrogen and phosphorus utilization efficiencies of corm and grain (Table S14 and Table 10). Furthermore, NUtE and PUtE of the both the grain and corm yields were not influenced by source of nitrogen. This shows neither manure nor urea treatment has a superior ability in producing grain and corm yields relative to plants’ tissue N and P. Moreover, intercropping increased nitrogen utilization efficiency which is corresponding to the conversion of absorbed N into corm yield per unit of nitrogen taken up by saffron plant.

**Table 10:** Means for nitrogen and phosphorus utilization efficiencies (NUtE and PUtE, respectively), nitrogen and phosphorus use efficiencies (NUE and PUE, respectively) for saffron corm and wheat grain and system nitrogen balance index (SNBI) all for the last (fourth) growing season.

Treatments	Utilization efficiency				Use efficiency				SNBI, kg ha^−1^
	NUtE, kg kg^−1^		PUtE, kg kg^−1^		NUE, kg kg^−1^		PUE, kg kg^−1^		
	Corm	Grain	Corm	Grain	Corm	Grain	Corm	Grain	
Irrigation regime									
40% ET_c_	65.8^a1^	36.1^a^	351.1^a^	277.0^a^	29.7^a^	16.7^a^	24.8^a^	14.6^a^	27.58^c^
60% ETc	64.5^a^	35.6^a^	330.2^a^	277.1^a^	36.0^a^	22.0^a^	30.4^a^	19.2^a^	21.93^c^
80% ETc	64.0^a^	36.1^a^	340.8^a^	285.3^a^	34.1^a^	20.1^a^	28.3^a^	17.6^a^	46.74^b^
100% ETc	65.8^a^	38.1^a^	363.6^a^	285.7^a^	32.3^a^	17.9^a^	27.0^a^	15.7^a^	64.1^a^
Source of nitrogen									
Manure	62.8^b^	36.0^a^	337.0^a^	270.3^a^	38.5^a^	18.0^a^	26.5^a^	12.4^b^	28.12^b^
Urea	67.3^a^	37.0^a^	355.8^a^	292.2^a^	27.6^b^	20.3^a^	28.7^a^	21.1^a^	52.05^a^
Cropping system									
Mono-cropping	63.2^b^	–	339.2^a^	–	36.6^a^	–	30.6^a^	–	59.11^a^
Intercropping	66.9^a^	–	353.7^a^	–	29.5^b^	–	24.7^b^	–	21.06^b^

*Means followed by similar letters in each column for each factor and each trait are not significantly different at 5% level of probability according to Tukey test.

### Nutrient use (yield) efficiency

There was no significant difference among irrigation regimes on both nitrogen and phosphorus use efficiencies for saffron corm and grain yield (Table S14 and Table 10). Moreover, no significant difference was observed between sources of nitrogen on grain NUE and corm PUE. However, urea treatment showed higher values of phosphorus use efficiency for grain yield due to the lower amounts of phosphorus (100 kg ha^−1^) supplied by triple superphosphate compared to the manure source of nitrogen which supplied an extra 46.0 kg ha^−1^ available phosphorus into the soil. It should be mentioned that all the manure and chemical urea treatments were supplied by 100.0 kg P ha^−1^, while the manure itself supplied an extra 46.0 kg P ha^−1^ into the soil. This fact makes the denominator of the PUE greater in manure treatments compared to urea, and smaller grain PUE in the manure treatments is the consequence. Since the nitrogen use efficiency is a measure of efficiency of input use, it can be concluded that manure can produce higher corm yield per unit of available N and higher saffron yield is a result (Table 1). Furthermore, mono-cropping showed higher values of nitrogen and phosphorus use efficiencies for saffron corm yield due to the nutrient competition of wheat plant.

### System N balance index (SNBI)

The last column in Table 10 shows the nitrogen balance index of the system (SNBI). This column actually indicates the difference between input nitrogen [the sum of N applied by sources of nitrogen and nitrogen added by irrigation water and rain N deposition (irrigation and rain water nitrogen concentration was 12.0 and 1.0 mg N L^−1^, respectively)] and the N removed (plant N uptake and leached nitrogen) from the soil. The values account for N losses other than N leaching, that is, N loss through denitrification, volatilization of NH_4_, gas emissions [NO_x_], plant senescence. Among the irrigation regimes, the lowest N balance value (the least nitrogen loss) was related to 60% ET_c_, from which the highest corm and saffron yield were harvested (Table 1). The highest value of SNBI was obtained from 100% ET_c_ from, which the highest leached N took place (Table 3). In addition, the results showed that the amount of SNBI in manure treatment was almost 46.0% lower that of chemical urea treatments. Moreover, intercropping reduced SNBI by 64.0% compared to sole cropping system. These recent findings indicate the preference of cow manure over chemical urea fertilizer and saffron–wheat intercropping over saffron sole cropping to improve a sustainable nitrogen and phosphorus system (soil, water, plant and atmosphere) of management in saffron production.

## Conclusion

The lowest and highest drainage water depth, seasonal nitrate concentration of the leachate and seasonal cumulative leached nitrate was observed in 40 and 100% ET_c_, respectively. Generally, the higher the gross water depth, the greater the drained water, its nitrate concentration, total leached nitrate resulted in higher risk of groundwater nitrate contamination, and lower crop(s) irrigation and economic water productivity, plant available nitrogen in root zone and crop(s) growth. The optimum irrigation regime was 60% ET_c_, where the highest saffron and wheat nutrient (nitrogen and phosphorus) uptake, nutrient (nitrogen and phosphorus) harvest indices, nutrient acquisition and use efficiencies, corm, saffron, and grain yields and lowest nitrogen loss (system nitrogen balance index) was achieved in this irrigation regime.

Moreover, manure as a slow-nitrogen-release fertilizer indicated 12.0, 42.0, 50.0 and 46.0% lower amounts of drained water, leachate nitrate nitrogen concentration, total leached nitrogen and system nitrogen balance index (implies nitrogen losses other than leaching), respectively, in comparison to the urea source of nitrogen which shows the lower risk of groundwater nitrate pollution. In addition, manure showed 9.0, 8.0 and 9.0% increase in the concentration of corm nitrogen, phosphorus and protein, respectively, in comparison to urea treatment. Saffron corm and stigma yields, irrigation and economic water productivities, corm nitrogen use efficiency and saffron-plant-nitrogen-acquisition efficiency in manure surpassed respectively, 21.0, 25.0, 20.0, 17.0, 39.0 and 49.0% the chemical source of nitrogen. A reversed outcome was achieved for grain and wheat plant as a whole. That is to say, irrigation and economic water productivity, nitrogen and phosphorus uptake, phosphorus-acquisition efficiency, nitrogen and phosphorus use efficiencies were higher for grain and wheat plant in chemical nitrogen application in contrast to manure source of nitrogen.

Furthermore, intercropping showed 10.0, 11.0, 23.0 and 64.0% lower amount of drained water, nitrate concentration in drainage water, seasonal leached nitrate and system nitrogen balance index, respectively compared to saffron sole cropping which reduces the risk of groundwaters nitrate contamination.

For all the experimental treatments, empirical regression models were derived for estimation of seasonal cumulative leached nitrate based on the seasonal cumulated drained water.

To cap it all, intercropping saffron with winter wheat, application of organic cow manure and adoption irrigation regime of 60% ET_c_ is an innovative system of saffron production which mitigates the risk of groundwater nitrate contamination. Saffron growers can make sustainable and clean use of the inter-row spaces of the saffron crop to grow winter wheat in order to obtain higher economic water productivity and lower groundwater pollution and it is highly recommended to a sustainable cropping system.

## Supplementary Information

Supplementary Information.

## Data Availability

All data generated or analyzed during the study are included in this published article or supplemental file attached to this article.
