# Supplementary material for: Nitrogen leaching and groundwater N contamination risk in saffron/wheat intercropping under different irrigation and soil fertilizers regimes
Source: Sci Rep. 2023 Apr 21;13:6587. doi: 10.1038/s41598-023-33817-5 (PMC10121562; doi:10.1038/s41598-023-33817-5)
Supplement: Supplementary file 1 — Supplementary Information. [file 41598_2023_33817_MOESM1_ESM.docx]

**Supplementary Information**

Table S1 Monthly climatic characteristics for the 1^st^ and 2^nd^ growing seasons

| **Year** | **Month** | **Mean daily of max. temp. (°C)** | **Mean daily of min. temp. (°C)** | **Mean daily of max. relative humidity (%)** | **Mean daily of min. relative humidity (%)** | **Wind speed (m s^-1^)** | **Sunshine (h d^-1^)** | **Rain  (mm month^-1^)** | **Evaporation from pan Class A (mm month^-1^)** |
| --- | --- | --- | --- | --- | --- | --- | --- | --- | --- |
| **2013-2014** | **Oct.** | 26.2 | 2.7 | 61.5 | 14.4 | 0.7 | 9.7 | 0.0 | 141.1 |
|  | **Nov.** | 17.3 | 2.3 | 74.4 | 23.3 | 0.5 | 6.4 | 72.5 | 72.4 |
|  | **Dec.** | 13.6 | -4.2 | 72.5 | 15.4 | 0.9 | 7.5 | 1.5 | 32.0 |
|  | **Jan.** | 7.8 | -7.1 | 77.2 | 36.5 | 0.4 | 5.1 | 105.5 | 28.0 |
|  | **Feb.** | 12.2 | -3.8 | 70.5 | 18.3 | 0.9 | 7.4 | 40.5 | 14.0 |
|  | **Mar.** | 17.6 | 2.0 | 69.7 | 18.7 | 1.2 | 7.4 | 29.5 | 102.4 |
|  | **Apr.** | 23.3 | 5.4 | 68.1 | 15.2 | 1.1 | 8.8 | 29.0 | 191.4 |
|  | **May.** | 28.1 | 8.8 | 57.7 | 13.3 | 1.0 | 9.8 | 1.0 | 258.5 |
|  | **Jun.** | 34.5 | 11.8 | 45.4 | 11.8 | 1.0 | 10.8 | 0.0 | 320.4 |
|  | **Jul.** | 36.4 | 15.9 | 41.4 | 13.6 | 0.9 | 10.6 | 0.0 | 344.6 |
|  | **Aug.** | 35.0 | 13.0 | 45.4 | 14.3 | 0.8 | 10.6 | 0.0 | 311.6 |
|  | **Sep.** | 32.2 | 9.6 | 49.9 | 22.4 | 0.9 | 10.8 | 0.0 | 251.9 |
|  | **Mean or sum** | **23.7** | **4.7** | **61.1** | **18.1** | **0.9** | **8.7** | **279.5** | **2068.3** |
| **2014-2015** | **Oct.** | 26.7 | 5.0 | 60.8 | 17.7 | 1.0 | 9.3 | 0.0 | 208.4 |
|  | **Nov.** | 15.8 | -0.7 | 70.5 | 18.2 | 0.8 | 7.5 | 77.5 | 46.0 |
|  | **Dec.** | 14.6 | -3.7 | 72.1 | 16.1 | 0.5 | 8.0 | 0.0 | 40.0 |
|  | **Jan.** | 13.9 | -2.3 | 66.0 | 18.1 | 1.1 | 7.7 | 23.0 | 38.9 |
|  | **Feb.** | 13.9 | 1.8 | 67.8 | 21.8 | 1.4 | 6.6 | 47.8 | 44.9 |
|  | **Mar.** | 16.9 | 3.3 | 67.6 | 21.4 | 1.2 | 6.5 | 73.5 | 98.9 |
|  | **Apr.** | 24.8 | 6.6 | 60.9 | 15.0 | 1.0 | 9.0 | 0.0 | 200.8 |
|  | **May.** | 28.9 | 9.3 | 51.9 | 12.7 | 1.0 | 9.9 | 10.0 | 215.9 |
|  | **Jun.** | 35.0 | 13.4 | 36.1 | 11.9 | 1.0 | 10.8 | 0.0 | 302.3 |
|  | **Jul.** | 35.3 | 16.5 | 38.5 | 14.0 | 0.9 | 10.3 | 0.0 | 322.8 |
|  | **Aug.** | 34.7 | 13.3 | 42.0 | 11.7 | 0.8 | 10.9 | 0.0 | 292.4 |
|  | **Sep.** | 30.7 | 10.4 | 51.1 | 13.7 | 0.9 | 8.9 | 1.0 | 212.0 |
|  | **Mean or sum** | **24.3** | **6.1** | **57.1** | **16.0** | **0.9** | **8.8** | **232.8** | **2023.4** |

Table S2 Monthly climatic characteristics for 3^rd^ and 4^th^ growing seasons

| **Year** | **Month** | **Mean daily of max. temp. (°C)** | **Mean daily of min. temp. (°C)** | **Mean daily of max. relative humidity (%)** | **Mean daily of min. relative humidity (%)** | **Wind speed (m s^-1^)** | **Sunshine (h d^-1^)** | **Rain  (mm month^-1^)** | **Evaporation from pan Class A (mm month^-1^)** |
| --- | --- | --- | --- | --- | --- | --- | --- | --- | --- |
| **2015-2016** | **Oct.** | 26.7 | 6.5 | 60.2 | 16.2 | 0.8 | 8.2 | 16.5 | 173.5 |
|  | **Nov.** | 17.0 | 1.2 | 72.4 | 16.0 | 0.9 | 10.2 | 68.0 | 53.6 |
|  | **Dec.** | 11.9 | -3.0 | 73.6 | 22.2 | 0.8 | 7.1 | 87.0 | 15.7 |
|  | **Jan.** | 12.1 | -4.1 | 73.9 | 19.0 | 0.7 | 6.4 | 64.5 | 14.1 |
|  | **Feb.** | 15.3 | -4.8 | 71.9 | 13.3 | 0.8 | 8.5 | 13.5 | 40.0 |
|  | **Mar.** | 18.6 | 2.5 | 66.3 | 16.6 | 1.3 | 7.6 | 20.5 | 129.6 |
|  | **Apr.** | 21.1 | 2.3 | 65.0 | 15.0 | 1.0 | 9.4 | 13.0 | 122.1 |
|  | **May.** | 29.8 | 8.6 | 53.0 | 10.6 | 1.0 | 10.9 | 6.0 | 249.6 |
|  | **Jun.** | 33.6 | 10.6 | 41.3 | 11.6 | 1.0 | 11.6 | 0.0 | 315.7 |
|  | **Jul.** | 37.1 | 15.7 | 43.0 | 13.2 | 1.0 | 10.8 | 0.0 | 337.3 |
|  | **Aug.** | 34.0 | 13.3 | 36.7 | 12.3 | 0.8 | 10.5 | 0.0 | 312.8 |
|  | **Sep.** | 32.7 | 9.8 | 48.0 | 13.7 | 1.0 | 10.3 | 0.0 | 267.0 |
|  | **Mean or sum** | **24.2** | **4.9** | **58.8** | **15.0** | **1.0** | **9.3** | **289.0** | **2030.9** |
| **2016-2017** | **Oct.** | 26.4 | 4.2 | 57.5 | 18.0 | 0.8 | 9.6 | 0.0 | 206.6 |
|  | **Nov.** | 19.9 | 0.5 | 58.7 | 19.4 | 0.7 | 6.8 | 11.3 | 105.3 |
|  | **Dec.** | 16.8 | 0.0 | 67.1 | 17.3 | 1.1 | 8.6 | 7.0 | 80.2 |
|  | **Jan.** | 14.4 | -1.8 | 66.1 | 18.5 | 1.3 | 7.1 | 27.5 | 38.9 |
|  | **Feb.** | 12.2 | -1.4 | 69.6 | 21.5 | 1.2 | 6.8 | 186.1 | 10.0 |
|  | **Mar.** | 16.8 | 1.4 | 70.1 | 17.8 | 1.2 | 6.6 | 107.0 | 205.2 |
|  | **Apr.** | 24.6 | 6.9 | 63.1 | 15.3 | 1.1 | 9.5 | 6.5 | 168.8 |
|  | **May.** | 29.4 | 9.5 | 59.8 | 12.9 | 0.8 | 10.0 | 22.5 | 189.4 |
|  | **Jun.** | 35.6 | 11.8 | 40.8 | 9.6 | 0.9 | 11.8 | 0.0 | 334.4 |
|  | **Jul.** | 36.1 | 15.1 | 37.5 | 12.7 | 0.8 | 10.6 | 0.0 | 349.8 |
|  | **Aug.** | 33.7 | 14.0 | 39.0 | 15.2 | 0.9 | 11.0 | 0.0 | 252.4 |
|  | **Sep.** | 31.7 | 8.8 | 48.5 | 11.2 | 0.7 | 10.1 | 0.0 | 202.1 |
|  | **Mean or sum** | **24.8** | **5.8** | **56.5** | **15.8** | **0.9** | **9.0** | **367.9** | **2143.1** |

Table S3 Averaged physico-chemical properties of the lysimeters’ soil

| Characteristic | Unit | Soil depth, cm | | |
| --- | --- | --- | --- | --- |
|  |  | 0-30 | 30-60 | 60-90 |
| Field capacity (FC) (-0.033 MPa) | cm^3^ cm^-3^ | 32 | 33 | 35 |
| Permanent wilting point (PWP) (-1.5 MPa) | cm^3^ cm^-3^ | 11 | 16 | 17 |
| Bulk density (BD) | g cm^-3^ | 1.31 | 1.37 | 1.43 |
| Saturated water content | cm^3^ cm^-3^ | 39 | - | - |
| Sand | % | 25 | 27 | 24 |
| Silt | % | 39 | 34 | 41 |
| Clay | % | 36 | 39 | 35 |
| Texture | - | CL^*^ | CL | CL |
| Electrical conductivity (EC) | dS m^-1^ | 0.69 | 0.55 | 0.71 |
| pH | - | 7.51 | 7.45 | 7.82 |
| Chloride (Cl^-^) | meq L^-1^ | 3.52 | 3.17 | 2.46 |
| Sodium (Na+) | meq L^-1^ | 2.72 | 1.51 | 2.18 |
| Calcium (Ca^2+^) | meq L^-1^ | 3.43 | 3.67 | 3.18 |
| Magnesium (Mg^2+^) | meq L^-1^ | 3.57 | 2.44 | 1.61 |
| Organic matter | % | 0.7 | 0.6 | 0.6 |
| Total Nitrogen | % | 0.020 | 0.021 | 0.023 |
| NO3-N | mg L^-1^ | 4.5 | 6.0 | 5.7 |
| NH4-N | mg L^-1^ | 4.0 | 4.3 | 4.2 |
| ^*^Clay loam | | | | |

Table S4 Chemical analysis of the irrigation water applied in the experiment

| Characteristic | Unit | Value | Characteristic | Unit | Value |
| --- | --- | --- | --- | --- | --- |
| Electrical conductivity | dS m^-1^ | 0.62 | Calcium (Ca^2+^) | meq L^-1^ | 3.9 |
| pH | - | 7.58 | Magnesium (Mg^2+^) | meq L^-1^ | 3.0 |
| Chloride (Cl^-^) | meq L^-1^ | 0.9 | Bicarbonate (HCO_3_^-^) | meq L^-1^ | 4.1 |
| Sodium (Na+) | meq L^-1^ | 0.62 | Sulfate (SO_4_^2-^) | meq L^-1^ | 2.5 |
| Phosphorus | meq L^-1^ | 0.003 | Nitrate-nitrogen (NO_3_^-^-N) | mg L^-1^ | 12 |
| Potassium (K^+^) | meq L^-1^ | 0.03 |  |  |  |

Table S5 Chemical analysis of the fermented cow manure

| Characteristic | value | Characteristic | value |
| --- | --- | --- | --- |
| EC, dS m^-1^ in 1:5 solution | 10.63 | Mg^2+^, meq l^-1^ in 1:5 solution | 17.50 |
| pH in 1:5 solution | 8.50 | K^+^, meq l^-1^ in 1:5 solution | 79.17 |
| Cl^-^, meq l^-1^ in 1:5 solution | 72.50 | Total phosphorus, % | 0.80 |
| Na+, meq l^-1^ in 1:5 solution | 20.73 | Total nitrogen, % | 2.10 |
| Ca^2+^, meq l^-1^ in 1:5 solution | 21.50 |  |  |

**Table S6 A summary of combined analysis of variance (ANOVA) for saffron, corm, grain and wheat straw yields for all the four growing seasons (reported from Abbasi and Sepaskhah, 2022)**

| Source of variation | Saffron yield | |  | Corm yield | |  | Wheat grain yield | |  | Wheat straw yield | |
| --- | --- | --- | --- | --- | --- | --- | --- | --- | --- | --- | --- |
|  | Df^2^ | Adj MS^3^ |  | df | Adj MS |  | df | Adj MS |  | df | Adj MS |
|  |  |  |  |  |  |  |  |  |  |  |  |
| R^1^ | 2 | 0.00366 |  | 2 | 1.847 |  | 2 | 0.0637 |  | 2 | 1.272 |
| I | 3 | 0.47955** | | 3 | 7.295 |  | 3 | 2.0602** |  | 3 | 5.124 |
| P | 1 | 0.24463 | | 1 | 48.766** |  | - | - |  | - | - |
| F | 1 | 0.51228* | | 1 | 39.058* |  | 1 | 6.6527** |  | 1 | 30.284** |
| Y | 3 | 0.07014** | | - | - |  | 2 | 1.1511* |  | 2 | 7.642* |
| I*P | 3 | 0.00748 |  | 3 | 3.053 |  | - | - |  | - | - |
| I*F | 3 | 0.00624 |  | 3 | 1.249 |  | 3 | 0.1884 |  | 3 | 1.387 |
| P*F | 1 | 0.0044 |  | 1 | 0.874 |  | - | - |  | - | - |
| I*P*F | 3 | 0.00605 |  | 3 | 0.159 |  | - | - |  | - | - |
| Error | 171 | 0.01191 |  | 30 | 4.291 |  | 60 | 0.248 |  | 60 | 2.345 |
| * and ** indicate statistically deferent at p= 0.05 and p= 0.01, respectively | | | | | | | | | | | |
| ^1^ R: Replication effect, I= Irrigation effect, P= Cropping system effect, F= Source of nitrogen effect, Y=year effect I*P=Irrigation –Cropping system interaction effect, … | | | | | | | | | | | |
| ^2^ df: Degree of freedom | | | | | | | | | | | |
| ^3^ Adj MS: Adjusted mean square | | | | | | | | | | | |

**Table S7 A summary of combined analysis of variance (ANOVA) for the gross irrigation water, drainage water and drainage water percentage**

| Source of variation | Degree of freedom | Adjusted mean square | | |
| --- | --- | --- | --- | --- |
|  |  | Gross irrigation, mm | Drainage water | Drainage percentage |
|  |  |  |  |  |
| R^1^ | 2 | 0 | 89 | 2.7 |
| I | 3 | 1300670 | 1165516** | 28870.5** |
| P | 1 | 0 | 11390** | 514.3** |
| F | 1 | 0 | 13611** | 597.5** |
| Y | 3 | 54903 | 5643** | 638** |
| I*P | 3 | 0 | 2515 | 117 |
| I*F | 3 | 0 | 2026 | 102 |
| P*F | 1 | 0 | 33430 | 1301.1 |
| I*P*F | 3 | 0 | 4341 | 157.7 |
| Error | 171 | 99 | 394 | 15.2 |
| * and ** indicate statistically deferent at p= 0.05 and p= 0.01, respectively ^1^ R: Replication effect, I= Irrigation effect, P= Cropping system effect, F= Source of nitrogen effect, Y=year effect I*P=Irrigation –Cropping system interaction effect, … | | | | |

**Table S8 A summary of combined analysis of variance (ANOVA) for the average seasonal nitrate concentration in drainage water, seasonal cumulative leached nitrate, and total leached nitrogen as a percentage of total available nitrogen in the applied fertilizer (urea or manure)**

| Source of variation | Degree of freedom | Adjusted mean square | | |
| --- | --- | --- | --- | --- |
|  |  | Leachate N concentration | Leached nitrate | Leached nitrate, % |
|  |  |  |  |  |
| R^1^ | 2 | 0.0089 | 0.3 | 0.22 |
| I | 3 | 7.1878** | 953.47** | 529.93** |
| P | 1 | 2.9303** | 56.24** | 36.96** |
| F | 1 | 64.424** | 360.26** | 432.99** |
| Y | 3 | 0.3504** | 4.79** | 2.31** |
| I*P | 3 | 0.2005 | 11.49 | 7.24 |
| I*F | 3 | 2.4585 | 98.77 | 120.59 |
| P*F | 1 | 0.0313 | 38.27 | 28.35 |
| I*P*F | 3 | 0.0362 | 5.97 | 4.6 |
| Error | 171 | 0.0162 | 0.33 | 0.16 |
| * and ** indicate statistically deferent at p= 0.05 and p= 0.01, respectively ^1^ R: Replication effect, I= Irrigation effect, P= Cropping system effect, F= Source of nitrogen effect, Y=year effect I*P=Irrigation –Cropping system interaction effect, … | | | | |

**Table S9 A summary of combined analysis of variance (ANOVA) for saffron and wheat grain irrigation water productivities (IWP_Saffron_ and IWP_Grain_, respectively), saffron, wheat grain and total economic water productivities (EWP_Saffron_, EWP_Grain_ and EWP_Total_, respectively)**

| Source of variation | IWP_Saffron_ | |  | EWP_Saffron_ | |  | EWP_Total_ | |  | IWP_Grain_ | |  | EWP_Grain_ | |
| --- | --- | --- | --- | --- | --- | --- | --- | --- | --- | --- | --- | --- | --- | --- |
|  | df^2^ | Adj. MS^3^ |  | df | Adj. MS |  | df | Adj. MS |  | Df | Adj MS |  | df | Adj MS |
|  |  |  |  |  |  |  |  |  |  |  |  |  |  |  |
| R^1^ | 2 | 0.004 |  | 2 | 0.0042 |  | 2 | 0.0064 |  | 2 | 0.0021 |  | 2 | 0.0003 |
| I | 3 | 0.480** | | 3 | 0.546** | | 3 | 0.5198** | | 3 | 0.1052** | | 3 | 0.016** |
| P | 1 | 0.245 | | 1 | 0.278** | | 1 | 0.1966** | | - | - |  | - | - |
| F | 1 | 0.512** | | 1 | 0.5829** | | 1 | 0.2827** | | 1 | 0.1264** | | 1 | 0.01857** |
| Y | 3 | 0.070** | | 3 | 0.0798** | | 2 | 0.0923** | | 2 | 0.0103 |  | 2 | 0.0015 |
| I*P | 3 | 0.007 |  | 3 | 0.0085 |  | 3 | 0.0008 |  | - | - |  | - | - |
| I*F | 3 | 0.006 |  | 3 | 0.0071 |  | 3 | 0.0085 |  | 3 | 0.0035 |  | 3 | 0.0005 |
| P*F | 1 | 0.004 |  | 1 | 0.005 |  | 1 | 0.0193 |  | - | - |  | - | - |
| I*P*F | 3 | 0.006 |  | 3 | 0.0068 |  | 3 | 0.0011 |  | - | - |  | - | - |
| Error | 171 | 0.012 |  | 171 | 0.0135 |  | 124 | 0.0137 |  | 60 | 0.005 |  | 60 | 0.00075 |
| * and ** indicate statistically deferent at p= 0.05 and p= 0.01, respectively | | | | | | | | | | | | | | |
| ^1^ R: Replication effect, I= Irrigation effect, P= Cropping system effect, F= Source of nitrogen effect, Y=year effect I*P=Irrigation –Cropping system interaction effect, … | | | | | | | | | | | | | | |
| ^2^ df: Degree of freedom | | | | | | | | | | | | | | |
| ^3^ Adj. MS: Adjusted mean square | | | | | | | | | | | | | | |

**Table S10 A summary of analysis of variance (ANOVA) for nitrogen and phosphorus concentration in the saffron and winter wheat organs for the last (fourth) growing season**

| Source of variation | Degree of freedom | |  | Adjusted mean square | | | | | | | | | | |
| --- | --- | --- | --- | --- | --- | --- | --- | --- | --- | --- | --- | --- | --- | --- |
|  | Saffron, corm, aboveground parts | Wheat, grain, straw |  | Nitrogen concentration | | | | |  | Phosphorus concentration | | | | |
|  |  |  |  | Saffron | |  | Wheat | |  | Saffron | |  | Wheat | |
|  |  |  |  | Corm | Aboveground parts |  | Grain | Straw |  | Corm | Aboveground parts |  | Grain | Straw |
|  |  |  |  |  |  |  |  |  |  |  |  |  |  |  |
| R^1^ | 2 | 2 |  | 0.015329 | 0.00417 |  | 0.0044 | 0.000003 |  | 0.02172 | 0.2457 |  | 0.11281 | 0.00107 |
| I | 3 | 3 |  | 0.015884 | 0.01863 |  | 0.04292 | 0.002622 |  | 0.07061 | 0.1744 |  | 0.06545 | 0.000611 |
| P | 1 | - |  | 0.044436* | 0.02308 |  | - | - |  | 0.14071 | 0.0003 |  | - | - |
| F | 1 | 1 |  | 0.121021** | 0.00426 |  | 0.00039 | 0.00065 |  | 0.27569* | 0.0102 |  | 0.16322 | 0.001258 |
| I*P | 3 | - |  | 0.0016 | 0.01325 |  | - | - |  | 0.00338 | 0.0107 |  | - | - |
| I*F | 3 | 3 |  | 0.000332 | 0.00885 |  | 0.00067 | 0.000655 |  | 0.02972 | 0.0276 |  | 0.00341 | 0 |
| P*F | 1 | - |  | 0.000207 | 0.00003 |  | - | - |  | 0.00482 | 0.0386 |  | - | - |
| I*P*F | 3 | - |  | 0.000228 | 0.01336 |  | - | - |  | 0.01765 | 0.0107 |  | - | - |
| Error | 30 | 14 |  | 0.008087 | 0.0143 |  | 0.01351 | 0.002357 |  | 0.05246 | 0.1185 |  | 0.04605 | 0.003779 |
| * and ** indicate statistically deferent at p= 0.05 and p= 0.01, respectively | | | | | | | | | | | | | | |
| ^1^ R: Replication effect, I= Irrigation effect, P= Cropping system effect, F= Source of nitrogen effect, I*P=Irrigation –Cropping system interaction effect, … | | | | | | | | | | | | | | |

**Table S11 A summary of analysis of variance (ANOVA) for protein concentration (PC) of saffron (corm and aboveground) and winter wheat (grain and straw) biomasses for the last (fourth) growing season**

| Source of variation | Degree of freedom | |  | Adjusted mean square | | | | |
| --- | --- | --- | --- | --- | --- | --- | --- | --- |
|  | For corm or aboveground part | For grain or straw |  | Protein concentration | | | | |
|  |  |  |  | Saffron | |  | Wheat | |
|  |  |  |  | Corm | Aboveground parts |  | Grain | Straw |
|  |  |  |  |  |  |  |  |  |
| R^1^ | 2 | 2 |  | 0.5988 | 0.1629 |  | 0.1717 | 0.00014 |
| I | 3 | 3 |  | 0.6205 | 0.7279 |  | 1.6764 | 0.10243 |
| P | 1 | - |  | 1.7358* | 0.9018 |  | - | - |
| F | 1 | 1 |  | 4.7274** | 0.1663 |  | 0.0151 | 0.02539 |
| I*P | 3 | - |  | 0.0625 | 0.5174 |  | - | - |
| I*F | 3 | 3 |  | 0.013 | 0.3458 |  | 0.0263 | 0.02557 |
| P*F | 1 | - |  | 0.0081 | 0.0013 |  | - | - |
| I*P*F | 3 | - |  | 0.0089 | 0.5219 |  | - | - |
| Error | 30 | 14 |  | 0.3159 | 0.5586 |  | 0.5277 | 0.09205 |
| * and ** indicate statistically deferent at p= 0.05 and p= 0.01, respectively | | | | | | | | |
| ^1^ R: Replication effect, I= Irrigation effect, P= Cropping system effect, F= Source of nitrogen effect, I*P=Irrigation –Cropping system interaction effect, … | | | | | | | | |
|  |  |  |  |  |  |  |  |  |

**Table S12 A summary of analysis of variance (ANOVA) for nutrient (nitrogen and phosphorus) uptake (kg ha^-1^) by the organs of saffron and winter wheat, as well as nitrogen and phosphorus harvest indices (NHI and PHI, respectively) [%] for saffron and wheat, all for the last (fourth) growing season**

| Source of variation | Degree of freedom | |  | Adjusted mean square | | | | | | | | | | | | | | | | |
| --- | --- | --- | --- | --- | --- | --- | --- | --- | --- | --- | --- | --- | --- | --- | --- | --- | --- | --- | --- | --- |
|  | Saffron, corm, Abvg.^2^ | Wheat, grain, straw |  | Nitrogen uptake | | | | |  | Phosphorus uptake | | | | |  | NHI | |  | PHI | |
|  |  |  |  | Saffron | |  | Wheat | |  | Saffron | |  | Wheat | |  | Saffron | Wheat |  | Saffron | Wheat |
|  |  |  |  | Corm | Abvg.^2^ |  | Grain | Straw |  | Corm | Abvg. |  | Grain | Straw |  |  |  |  |  |  |
|  |  |  |  |  |  |  |  |  |  |  |  |  |  |  |  |  |  |  |  |  |
| R^1^ | 2 | 2 |  | 23 | 0.6 |  | 35.5 | 42.7 |  | 0.5 | 0.8 |  | 4.1 | 0.38 |  | 2.7 | 27.0 |  | 32.5 | 65.2 |
| I | 3 | 3 |  | 468.7* | 6.5 |  | 325.1 | 59.4 |  | 17.6* | 2.4 |  | 6.5* | 0.23 |  | 36.7 | 18.4 |  | 11.5 | 29.2 |
| P | 1 | - |  | 2564.6** | 311.0** |  | - | - |  | 85.0** | 9.0** |  | - | - |  | 1.1 | - |  | 8.74 | - |
| F | 1 | 1 |  | 2633.4** | 166.7** |  | 753.1* | 180.3 |  | 76.8** | 7.1* |  | 7.9* | 1.65 |  | 15.3 | 25.9 |  | 17.4 | 0.79 |
| I*P | 3 | - |  | 136.6 | 7.6 |  | - | - |  | 4.2 | 0.1 |  | - | - |  | 0.6 | - |  | 4.2 | - |
| I*F | 3 | 3 |  | 31.9 | 4.9 |  | 12.1 | 6.5 |  | 0.2 | 0.5 |  | 0.06 | 0.00 |  | 7.1 | 7.5 |  | 21.3 | 0.2 |
| P*F | 1 | - |  | 43.5 | 2.1 |  | - | - |  | 2.7 | 0.2 |  | - | - |  | 1.5 | - |  | 0.01 | - |
| I*P*F | 3 | - |  | 9.2 | 21.3 |  | - | - |  | 0.3 | 0.3 |  | - | - |  | 15.2 | - |  | 6.9 | - |
| Error | 30 | 14 |  | 133.2 | 13.3 |  | 97.7 | 61.4 |  | 5.4 | 1.0 |  | 1.7 | 0.87 |  | 19.1 | 66.4 |  | 42.1 | 62.62 |
| * and ** indicate statistically deferent at p= 0.05 and p= 0.01, respectively | | | | | | | | | | | | | | | | | | | | |
| ^1^ R: Replication effect, I= Irrigation effect, P= Cropping system effect, F= Source of nitrogen effect, I*P=Irrigation –Cropping system interaction effect, … | | | | | | | | | | | | | | | | | | | | |
| ^2^ Abvg.: Aboveground biomass | | | | | | | | | | | | | | | | | | | | |

**Table S13 A summary of analysis of variance (ANOVA) for nitrogen and phosphorus acquisition efficiencies (NAE and PAE, respectively) [%] for saffron corms, wheat grain and the whole plants (saffron and wheat), all for the last (fourth) growing season**

| Source of variation | Degree of freedom | |  | Adjusted mean square | | | | | | | | | | |
| --- | --- | --- | --- | --- | --- | --- | --- | --- | --- | --- | --- | --- | --- | --- |
|  |  |  |  | Acquisition (uptake) efficiency for yield | | | | |  | Acquisition (uptake) efficiency for the whole plant | | | | |
|  | For saffron or corm | For wheat or grain |  | NAE | |  | PAE | |  | NAE | |  | PAE | |
|  |  |  |  | Corm | Grain |  | Corm | Grain |  | Saffron | Wheat |  | Saffron | Wheat |
|  |  |  |  |  |  |  |  |  |  |  |  |  |  |  |
| R^1^ | 2 | 2 |  | 16.39 | 17.35 |  | 0.106 | 1.5258 |  | 18.71 | 37.53 |  | 0.042 | 0.94 |
| I | 3 | 3 |  | 229.47* | 156.3* |  | 6.382* | 2.3492* |  | 254.54* | 323.7* |  | 11.503** | 3.14** |
| P | 1 | - |  | 1289.68** | - |  | 29.037** | - |  | 2340.39** | - |  | 51.063** | - |
| F | 1 | 1 |  | 2998.3** | 84.47 |  | 0.087 | 18.1195** |  | 4868.76** | 110.78 |  | 0.625 | 64.14** |
| I*P | 3 | - |  | 65.66 | - |  | 1.49 | - |  | 98.79 | - |  | 2.185 | - |
| I*F | 3 | 3 |  | 14.8 | 2.51 |  | 0.265 | 0.0802 |  | 12.94 | 2.1 |  | 0.119 | 0.14 |
| P*F | 1 | - |  | 50.76 | - |  | 0.06 | - |  | 81.29 | - |  | 0.087 | - |
| I*P*F | 3 | - |  | 3.07 | - |  | 0.148 | - |  | 22.97 | - |  | 0.407 | - |
| Error | 30 | 14 |  | 67.44 | 45.31 |  | 1.933 | 0.6027 |  | 87.51 | 69.7 |  | 2.466 | 0.46 |
| * and ** indicate statistically deferent at p= 0.05 and p= 0.01, respectively | | | | | | | | | | | |  |  |  |
| ^1^ R: Replication effect, I= Irrigation effect, P= Cropping system effect, F= Source of nitrogen effect, I*P=Irrigation –Cropping system interaction effect, … | | | | | | | | | | | | | | |

**Table S14 A summary of analysis of variance (ANOVA) for nitrogen and phosphorus utilization efficiencies (NUtE and PUtE, respectively) and nitrogen and phosphorus use efficiencies (NUE and PUE, respectively) for saffron corm and wheat grain, all for the last (fourth) growing season**

| Source of variation | Degree of freedom | |  | Adjusted mean square | | | | | | | | | | |
| --- | --- | --- | --- | --- | --- | --- | --- | --- | --- | --- | --- | --- | --- | --- |
|  |  |  |  | Utilization efficiency | | | | |  | Use efficiency | | | | |
|  | For saffron or corm | For wheat or grain |  | NUtE | |  | PUtE | |  | NUE | |  | PUE | |
|  |  |  |  | Corm | Grain |  | Corm | Grain |  | Corm | Grain |  | Corm | Grain |
|  |  |  |  |  |  |  |  |  |  |  |  |  |  |  |
| R^1^ | 2 | 2 |  | 61.45 | 24.18 |  | 1808 | 346 |  | 23.61 | 10.68 |  | 16 | 9.11 |
| I | 3 | 3 |  | 10.78 | 7.55 |  | 2449 | 142 |  | 86.32 | 32.67 |  | 67.84 | 25.41 |
| P | 1 | - |  | 166.8* | - |  | 2532 | - |  | 603.13** | - |  | 414.95** | - |
| F | 1 | 1 |  | 245.98 | 6.16 |  | 4257 | 2859 |  | 1419.39** | 31.15 |  | 58.72 | 455.41** |
| I*P | 3 | - |  | 3.38 | - |  | 287 | - |  | 36.19 | - |  | 28.2 | - |
| I*F | 3 | 3 |  | 4.56 | 2.42 |  | 1922 | 98 |  | 13.28 | 0.48 |  | 15.45 | 2.32 |
| P*F | 1 | - |  | 6.69 | - |  | 68 | - |  | 24.6 | - |  | 0 | - |
| I*P*F | 3 | - |  | 17.43 | - |  | 327 | - |  | 1.24 | - |  | 3.13 | - |
| Error | 30 | 14 |  | 35.55 | 23.28 |  | 1396 | 1647 |  | 54.42 | 14.51 |  | 35.58 | 12.51 |
| * and ** indicate statistically deferent at p= 0.05 and p= 0.01, respectively | | | | | | | | | | | |  |  |  |
| ^1^ R: Replication effect, I= Irrigation effect, P= Cropping system effect, F= Source of nitrogen effect, Y=year effect I*P=Irrigation –Cropping system interaction effect, … | | | | | | | | | | | | | | |

| 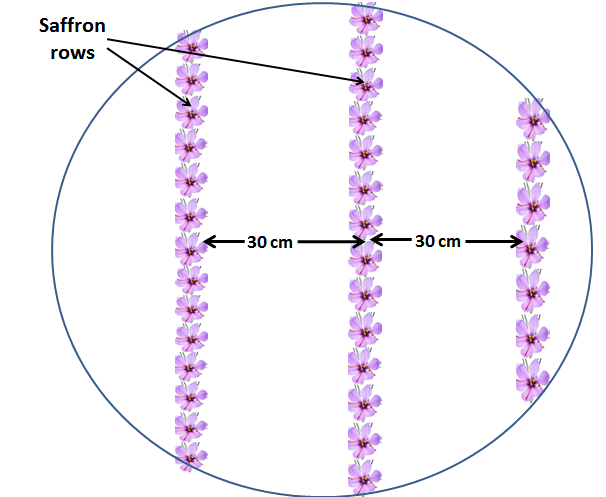 |
| --- |
| **A** |
| 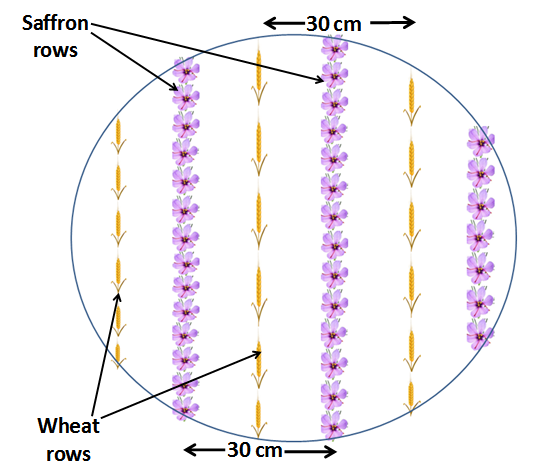 |
| **B** |

Fig. S1 A schematic plan view of cropping system in a lysimeter: a) mono cropping b) intercropping system.
